# BPEI-Based N-Doped Carbon Dots with Sensitive and Selective Cu^2+^ Ion-Sensing Ability

**DOI:** 10.3390/mi16111275

**Published:** 2025-11-13

**Authors:** Sahin Demirci, Jorge H. Torres, Nurettin Sahiner

**Affiliations:** 1 Department of Food Engineering, Faculty of Engineering, Istanbul Aydin University, Florya Halit Aydin Campus, Istanbul 34153, Turkey; 2 Department of Bioengineering, U.A. Whittaker College of Engineering, Florida Gulf Coast University, Fort Myers, FL 33965, USA; 3 Department of Chemical Engineering, Faculty of Engineering, Canakkale Onsekiz Mart University, Terzioglu Campus, Canakkale 17100, Turkey

**Keywords:** fluorescence quenching, metal ion sensor, selective Cu^2+^ sensing, environmental water, Cu^2+^ sensor

## Abstract

In this research, we examined the potential sensor characteristics of branched polyethyleneimine (BPEI)-derived carbon dots (CDs) synthesized using BPEI as a nitrogen source and citric acid (CA) as a carbon source, specifically for the recognition of various metal ions. Among the BPEI CDs produced with different amounts of BPEI to CA BPEI:CA ratios of 0.5:1, 1:1, and 2:1 *w*/*w*, named as BPEI^0.5^ CD, BPEI^1^ CD, and BPEI^2^ CD, respectively. The BPEI^0.5^ CD, which contains the least BPEI, exhibited the highest fluorescence intensity: 50,300 a.u. in a 0.6 mg/mL solution were recorded as λ_em_: 420 nm at λ_ex_: 360 nm and 600 V PMT voltage with 5 nm of slit width for both excitation and emission. We investigated the fluorescence variations in BPEI CD-based CDs in 2 mL solutions containing Cd^2+^, Co^2+^, Cu^2+^, Ni^2+^, and Pb^2+^ metal ions at various concentrations. Amongst these metal ions, the most pronounced sensitivity was noted for Cu^2+^ ions with a limit of detection (LOD) value of 0.39 ppm. For BPEI CDs created with BPEI:CA ratios of 0.5:1, 1:1, and 2:1 *w*/*w*, the sensitivity to Cu^2+^ ions increased with a higher BPEI ratio, with a LOD value of 0.30 ppm recorded for BPEI^2^ CDs. Moreover, Cu^2+^ ion solutions were prepared from various salts, including chloride, acetate, nitrate, and sulfate; aside from some fluorescence variation observed for BPEI^0.5^ CDs, no significant difference in BPEI CD fluorescence change was observed with the use of the various salt solutions of Cu^2+^ ion. In quenching experiments conducted on mixtures of Cd^2+^, Co^2+^, Cu^2+^, Ni^2+^, and Pb^2+^ metal ions with Cu^2+^, it was noted that BPEI CDs displayed selectivity for Cu^2+^ ions. Furthermore, the structures of BPEI CDs have been effectively utilized in real water samples, such as tap water and seawater, demonstrating a quenching capability of over 65% in the presence of 50 ppm Cu^2+^ ions.

## 1. Introduction

The most remarkable characteristics of carbon dots (CDs) encompass fluorescence, solubility in water, chemical stability, and minimal cytotoxicity, which have facilitated their application across a diverse array of fields [1,2,3,4]. The photoluminescence properties of these CDs are attributed to surface defects, quantum confinement phenomena, and the existence of various oxygen-containing functional groups, including hydroxyl, carbonyl, and carboxyl groups, as well as various other functionalities [5,6,7]. The scope of their applications is further broadened through doping with different heteroatoms [8,9]. Among these, nitrogen is the most frequently utilized dopant heteroatom [10]. Nitrogen-doped carbon dots (N-CDs) are produced by integrating nitrogen atoms into the carbon framework or surface, resulting in notable alterations in quantum yield, photostability, and electronic characteristics [11,12]. N-CDs have been extensively employed in metal ion sensing, biological imaging, catalysis, and optoelectronic applications [13,14,15]. N-CDs demonstrate enhanced sensing capabilities for metal ions compared to neat CDs, attributable to the incorporation of nitrogen atoms into the carbon framework [16,17,18,19]. This modification elevates surface reactivity, electron density, and photoluminescence efficiency [16,17]. The introduction of nitrogen atoms generates electron-rich regions that alter the electronic structure, thereby promoting robust coordination interactions with metal ions [20,21]. The sensing mechanisms employed by N-CDs primarily encompass photoinduced electron transfer (PET), fluorescence resonance energy transfer (FRET), and the formation of static complexes between metal ions and nitrogen-containing functional groups (–NH_2_, –C=N, –C–N) [22,23]. Notably, transition metal ions such as Cu^2+^, Fe^3+^, and Hg^2+^ effectively diminish the fluorescence of N-CDs by accepting electrons from the excited carbon nucleus, which results in nonradiative recombination and a decrease in emission intensity [6,18,24]. The nature and concentration of the nitrogen dopant play a crucial role in determining selectivity and sensitivity; an increased presence of pyridinic and graphitic N typically enhances electron-donating capacity and binding affinity towards metal ions [25,26,27,28]. As a result, N-CDs are positioned as effective, cost-efficient, and environmentally sustainable fluorescent probes for the selective and quantitative identification of hazardous metal ions in both environmental and biological contexts [17,21,25,27].

Copper (Cu) is a vital trace metal; however, elevated levels in aquatic ecosystems or drinking water can present risks to both ecological systems and human health [29,30]. The World Health Organization (WHO) stipulates that the maximum permissible concentration of Cu^2+^ ions in drinking water is 2 mg/L, a limit established based on gastrointestinal effects noted in sensitive populations [31]. In contrast, the U.S. Environmental Protection Agency (EPA) has determined a slightly lower threshold of 1.3 mg/L as part of the Lead and Copper Rule [32]. Consequently, it is crucial to keep Cu^2+^ ion concentrations within these established limits to avert environmental toxicity and negative health impacts on humans. An excess of Cu^2+^ ions in the human body is linked to various pathological conditions, primarily due to their potent redox activity and capacity to promote the formation of reactive oxygen species [33,34,35,36]. The most notable condition associated with this excess is Wilson disease, which arises from a genetic mutation in the ATP7B copper-transporting ATPase [37,38]. This defect results in the accumulation of copper in the liver, brain, and cornea, leading to severe consequences such as liver failure, neuropsychiatric issues, and the distinctive Kayser–Fleischer rings [39,40,41]. Excessive dietary intake or environmental exposure to Cu can lead to nongenetic Cu toxicity, which may result in oxidative stress, damage to the liver and kidneys, hemolytic anemia, and gastrointestinal issues [42,43,44]. Furthermore, the dysregulation of copper homeostasis has been associated with the development of neurodegenerative diseases such as Alzheimer’s and Parkinson’s, where abnormal copper levels contribute to protein misfolding and oxidative damage to neurons [45,46,47]. Consequently, it is crucial to maintain copper homeostasis for cellular redox stability and overall physiological well-being. In healthy adults, the total serum (or plasma) copper concentration generally falls within the range of approximately 10 to 25 μmol/L (≈0.6–1.6 mg/L) [48].

In this research, branched polyethyleneimine (BPEI) with a molecular weight of 25,000 g/mol was utilized to synthesize N-doped CD structures, which have been thoroughly characterized and examined for their biomedical properties in earlier investigations [49,50]. These structures were referred to as BPEI-based CDs, and their capabilities as metal ion sensors were explored. The BPEI-based CDs were synthesized from a solution containing varying concentrations of BPEI as N source and citric acid as a carbon source via a hydrothermal method in a Teflon-lined autoclave at a temperature of 250 °C. To assess their potential for sensor applications, the changes in fluorescence intensity of the BPEI CDs in the presence of metal ions such as Cd^2+^, Co^2+^, Cu^2+^, Ni^2+^, and Pb^2+^ were recorded. Additionally, the influence of the quantity of PEI, used as a precursor, along with different salts of Cu^2+^ ions including chloride, acetate, nitrate, and sulfate, were examined. Sensitivity studies, including the limit of detection (LOD), and selectivity assessments for BPEI-CDs in relation to Cu^2+^ ions were also carried out. Moreover, the potential sensing capabilities of the synthesized BPEI-CD structures against Cu^2+^ ions in real water samples, including tap water and seawater, were investigated.

## 2. Materials and Methods

### 2.1. Materials

Branched poly(ethyleneimine) (BPEI, Mn:25,000, 50% in water, Sigma Aldrich, St. Louis, MO, USA) was used as received. Citric acid (CA, 99.5%, Carlo Erba, Emmendingen, Germany) was used as carbon source for the synthesis of CDs. Copper acetate monohydrate (Cu(CH_3_COO)_2_.H_2_O, 98%, Sigma Aldrich), copper chloride anhydrous (CuCl_2_, 99%, Kimetsan, Ankara, Turkiye), copper nitrate hexahydrate (Cu(NO_3_)_2_.6H_2_O, 98%, Fluka, Buchs, Switzerland), and copper sulfate pentahydrate (Cu(SO_4_)_2_.5H_2_O, 99%, Sigma Aldrich) metal salts were used as sources of Cu(II) ions. Cobalt chloride hexahydrate (CoCl_2_.6H_2_O, 98%; Aldrich, Wien Austria), nickel chloride hexahydrate (NiCl_2_.6H_2_O, 98%; Acros, Renningen, Germany), Cadmium chloride hydrate (CdCl_2_.xH_2_O, 98%, Fluka), and lead chloride (PbCl_2_, 98%, Sigma Aldrich) were used as other metal ion sources. All experiments were performed in distilled water.

### 2.2. Synthesis and Characterization of BPEI CDs

In the preparation of BPEI-based carbon dots (CDs), various ratios of branched polyethyleneimine (BPEI) were utilized together with citric acid (CA) in proportions such as 0.5:1, 1:1, and 2:1 *w*/*w* (BPEI:CA ratios) separately. In the synthesis of BPEI CDs, 0.25, 0.5, and 1 g of BPEI (Mn 25,000 g/mol, Sigma Aldrich) were incorporated into 20 mL of water containing 0.5 g of citric acid (CA, 99%, Carlo Erba). Subsequently, these mixtures were stirred at ambient temperature for 5 min to ensure the complete dissolution of all constituents. Ultimately, the solutions were transferred into a Teflon-lined autoclave and subjected to heating in a furnace. The furnace was set to reach 250 °C at a heating rate of 10 °C/min and maintained at this temperature for 4 h. Next, the prepared BPEI CDs were placed into a dialysis membrane with a molecular weight cutoff: 12,000 Da (Sigma-Aldrich) and washed in 500 mL of water 3 times by changing the wash water every 2 h to remove unreacted precursors. After that, the resultant BPEI CDs precipitated upon exposure to an excess of acetone, resulting in a sticky consistency. The synthesized BPEI-based CDs were designated as BPEI^0.5^, BPEI^1^, and BPEI^2^ CDs, corresponding to the ratios of BPEIs to CA.

To characterize the prepared BPEI CDs, transmission electron microscopy (TEM, HT7800, Hitachi, Tokyo, Japan), Fourier transform infrared (FT-IR, Nicolet iS10, Thermo Fisher Scientific, Waltham, MA, USA) spectroscopy and X-ray diffraction (XRD) analyses were conducted. TEM images were acquired under vacuum at an operating voltage of 100 kV. The FT-IR spectra of the synthesized BPEI CDs were recorded in the wavelength range of 4000 to 650 cm^−1^ with a resolution of 4 cm^−1^ utilizing the ATR technique. XRD patterns of the BPEI CDs were obtained using a X’Pert Pro MPD diffractometer (PANalytical, Almelo, The Netherlands), which was equipped with CuKα radiation and the X’Celerator detector for the diffracted beam. The XRD data were gathered in a Bragg–Brentano (*θ*/*θ*) vertical geometry, functioning in flat reflection mode, over a range of 10° to 80° (2θ) in increments of 0.02° 2θ, with a counting time of 1 s per step. The X-ray tube operated at 45 kV and 40 mA, and a 1/2° divergence slit, a 0.04 rad Soller slit, and a 10 mm fixed mask were positioned in the pathway of the incident beam. The High Score Plus (v.4.6.0) software facilitated peak identification and automated search-match for the analysis of the diffraction patterns.

### 2.3. Optical Properties of BPEI CDs

The optical characteristics of BPEI CDs were examined through UV-Vis spectroscopy (using a Spectrum UV spectrometer) and Fluorescence Spectroscopy (Lumina, Thermo, Victor, NY, USA) at ambient temperature. The concentrations of the BPEI CD solutions were set to 0.015 mg/mL to achieve a satisfactory UV-Vis spectrum, which was recorded over a wavelength range of 190–500 nm. Conversely, the concentrations of the same BPEI CD solutions were increased to 0.6 mg/mL [50] for fluorescence spectrometric analysis using a wavelength range of 360–640 nm with 5 nm of slit width for both excitation and emission. The PMT voltages were calibrated to 600 V for all measurements, and each measurement was conducted in triplicate. The results were compiled into averages of three measurements accompanied by standard deviations.

### 2.4. Quenching Properties of BPEI CDs

The changes in the fluorescence characteristics of the synthesized BPEI-based CDs occurring in the presence of different metal ions were examined to assess their potential use in sensor applications. Initially, the synthesized BPEI^1^ CD structures were dispersed in solutions containing metal ions at varying concentrations from 3.12 to 1000 ppm. The metal ions employed included chloride salts of Co^2+^, Ni^2+^, Cu^2+^, Cd^2+^, and Pb^2+^.

Subsequently, the change in the fluorescence intensity of 0.6 mg/mL BPEI^0.5^ and BPEI^2^ CD solutions were examined in the presence of solutions containing different concentrations of Cu^2+^ ions (ranging from 3.12 to 1000 ppm). Consequently, the quantity of BPEI employed in the fabrication of BPEI-based CD structures was evaluated in terms of its influence on the CDs metal-sensing ability.

Moreover, the changes in the fluorescence characteristics of the synthesized BPEI-based CD structures were examined in the presence of 50 ppm solutions of various salts of the Cu^2+^ ion, e.g., acetate, nitrate, and sulfate, with BPEI-based CD solutions at a concentration of 0.6 mg/mL. These particular tests allowed the evaluation of the impact of different anions on the CDs’ Cu^2+^-sensing properties.

For every measurement conducted, the overall volume of each solution was maintained at 2 mL. The fluorescence assessments were carried out across an emission wavelength spectrum ranging from 360 to 640 nm, with the excitation wavelength fixed at 360 nm, 5 nm of slit width for both excitation and emission, a PMT voltage of 600 V, and a BPEI CD concentration of 0.6 mg/mL. Each test was performed in triplicate, and the findings were compiled as averages along with their corresponding standard deviations.

### 2.5. Selectivity of BPEI CDs to Cu^2+^ Ions

To examine the selectivity of BPEI^1^ CDs for Cu^2+^ ion, fluorescence measurements were conducted in mixed solutions of the metal ions detailed in Section 2.3, maintaining a final concentration of 50 ppm. The mixed metal ion solutions employed included Cu^2+^-Co^2+^, Cu^2+^-Ni^2+^, Cu^2+^-Cd^2+^, Cu^2+^-Pb^2+^, and a comprehensive solution of Cu^2+^-Co^2+^-Ni^2+^-Cd^2+^-Pb^2+^ that contained all the specified metal ions. The total volume of each solution was 2 mL. The fluorescence measurements were performed over an emission wavelength range of 360–640 nm, with an excitation wavelength set at 360 nm, 5 nm of slit width for both excitation and emission, a PMT voltage of 600 V, and a BPEI^1^ CD concentration of 0.6 mg/mL. Each test was performed in triplicate, and the findings were recorded as averages along with their corresponding standard deviations.

### 2.6. Quenching Ability of BPEI CDs in the Presence of Cu^2+^ Ions in Real Waters

The changes in fluorescence intensity of BPEI^1^ CDs upon exposure to Cu^2+^ ions were investigated in real water samples. To facilitate this, solutions containing Cu^2+^ ions were prepared at a final concentration of 50 ppm in both Dardanelles (Phosphorous–Çanakkale) seawater and tap water. A total volume of 2 mL of the metal ion solution was employed. For the fluorescence measurements, as with the other tests, the emission wavelength range was measured between 360 and 640 nm, with an excitation wavelength of 360 nm, 5 nm slit width for both excitation and emission, a PMT voltage of 600 V, and a BPEI^1^ CD concentration of 0.6 mg/mL. Each test was performed in triplicate, and the findings were reported as averages along with their corresponding standard deviations.

## 3. Results and Discussion

### 3.1. Characterization and Optical Properties of BPEI CDs

**Figure 1:**
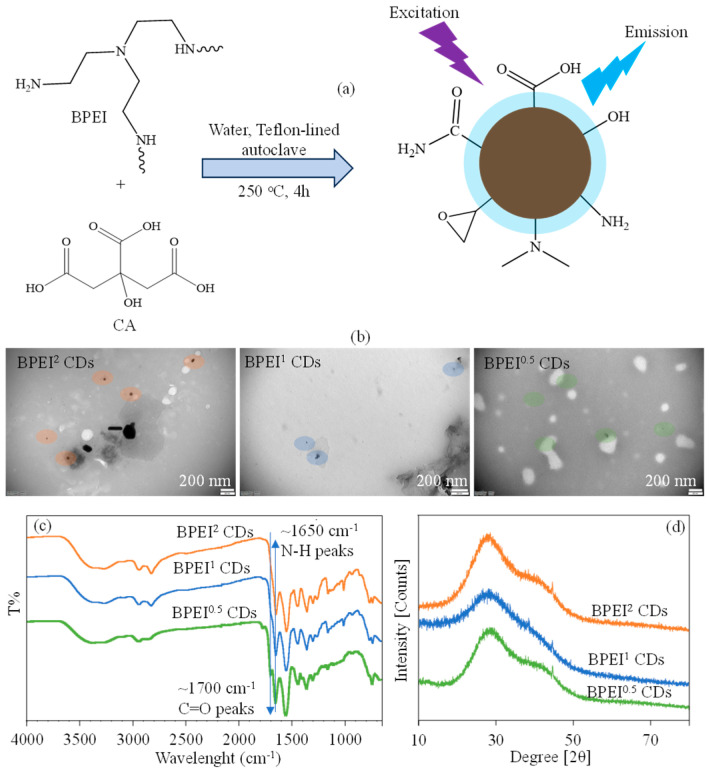
(**a**) The schematic presentation of synthesis of BPEI based CDs via hydrothermal method, (**b**) TEM images, (**c**) FT-IR spectrum, and (**d**) XRD pattens of BPEI based CDs.

The synthesis and characterization of BPEI-based CDs have been reported by our group in earlier studies [49,50]. As illustrated in Figure 1a, the synthesis of PEI-based CDs involves the use of a carbon source such as citric acid (CA), while BPEI acts as the nitrogen (N) source in a one-step hydrothermal method synthesis technique [49,50]. The formation of CDs is promoted by the generation of amide bonds through the thermal dehydration of ammonium and carboxylate groups, which arise from the covalent interaction between excess BPEI molecules and CA molecules [51,52]. A proposed mechanism for the CD synthesis suggests the development of a polymeric network from two precursor molecules abundant in functional groups, followed by carbonization at elevated temperatures [19,52,53]. The resultant BPEI CDs possess a range of functional groups, including hydroxyl, epoxide, carboxylic acid, and amine groups, as reported in the literature [25,26,27]. BPEI CDs are generated through a concurrent carbonization process that includes the integration of PEI structure into the reaction. The gravimetric yields for BPEI CDs, derived from PEI and CA at weight ratios of 0.5:1, 1:1, and 2:1, were determined and reported earlier [49,50] and found as 72 ± 4%, 83 ± 6%, and 89 ± 6%, respectively.

The TEM images of BPEI^2^, BPEI^1^, and BPEI^0.5^ CDs were also given in Figure 1b, respectively, and the visible sizes of CDs were decreased with the increase in CA ratio, and this result is in accordance with the literature [49,50]. The dimensions of BPEI CDs at a 1:1 ratio were reported to be around 54 ± 5 nm [50], and another investigation suggested that an increase in PEI ratio bestowed particles with larger sizes, whereas a decrease in its ratio led to smaller sizes [49]. In the FT-IR analysis in Figure 1c, it has been noted that as the CA ratio in BPEI CDs increases, the intensity of the C=O peaks, observed at about 1700 cm^−1^ also rises as anticipated, while the N-H peaks around 1650 cm^−1^ similarly increase with the increase in BPEI ratios [49]. It has been established that the increase in the BPEI precursor contributes to higher levels of amino residues in BPEI CDs. Furthermore, an XRD analysis of BPEI CDs was performed for the samples at 0.5:1, 1:1, and 2:1 weight ratios of PEI and CA, as given in Figure 1d. The peaks around 2θ = 28° was assigned to carbon phase, and the crystallite sizes of the BPEI CDs were determined to be 1.0, 1.4, and 1.0 nm, respectively, is in accord with the literature [49].

**Figure 2:**
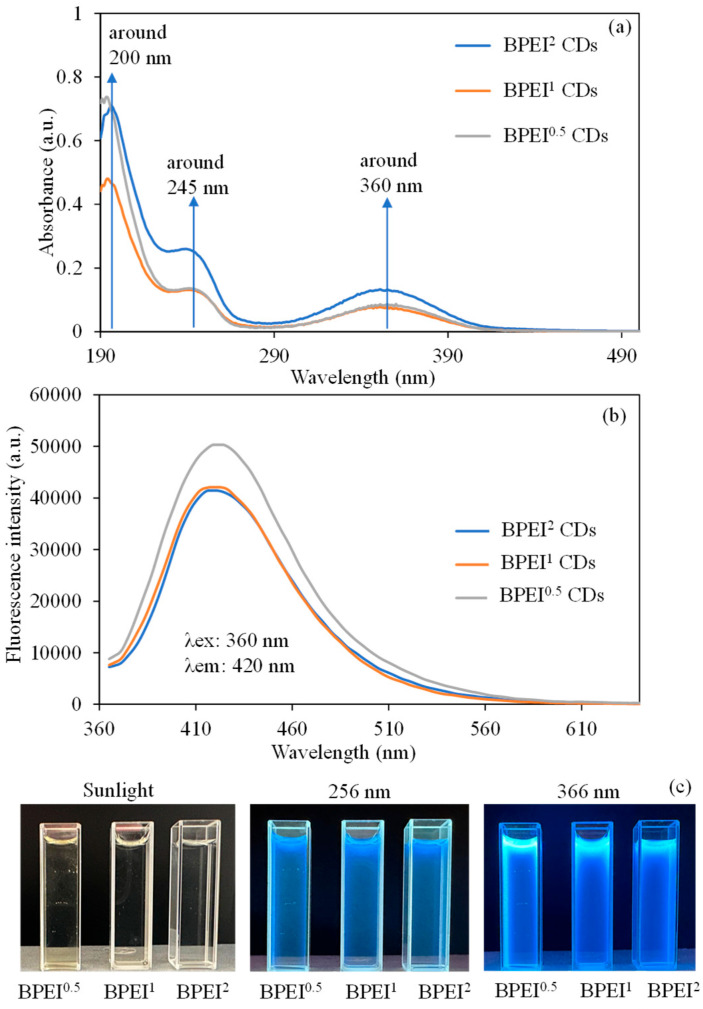
(**a**) UV-Vis spectrum, (**b**) fluorescence spectrum, and (**c**) digital camera images of BPEI based CDs.

The absorbance UV-Vis spectra of BPEI CDs, which were synthesized using weight ratios of 0.5:1, 1:1, and 2:1 for PEI and CA, are presented in Figure 2a. As depicted in Figure 1a, the significant absorption peak around 200 nm is linked to the π-π* transition of the surface states related to C=N and C=C bonds found in all variations in BPEI-CD. Additionally, the subtle peak noted at 245 nm is connected to the π-π* transition of surface states that include C=N groups, which is observable in the CQ points of BPEI-CDs [54]. The small peak at 360 nm is attributed to the n-π* transition of carbonyl groups and surface functional groups present on the nitrogen-rich surfaces of BPEI CDs [55,56].

A graph illustrating the fluorescence characteristics of the three synthesized BPEI CDs at 0.6 mg/mL concentrations is presented in Figure 2b. Consequently, a higher BPEI ratio resulted in a reduction in fluorescence properties, whereas an increased CA ratio led to an enhancement of fluorescence properties. During the analysis, under illumination at an excitation wavelength of 360 nm, the fluorescence intensities recorded for BPEI^0.5^, BPEI^1^, and BPEI^2^ CDs were approximately 50,300, 44,500, and 41,400 a.u., respectively, at an emission wavelength of 420 nm. The digital camera images of BPEI^0.5^, BPEI^1^, and BPEI^2^ CD solutions at 0.6 mg/mL concentrations were also given in Figure 2c under sunlight, 256 nm, and 366 nm UV-light. An overabundance of PEI content adds an excessive number of nitrogen functionalities (–NH_2_, –CONH–, –C–N–, etc.) to the surface of the CD [25,27]. This alteration diminishes the conjugated π-sites within the carbon core that are essential for fluorescence, leading to a reduction in the size of sp^2^ domains and consequently resulting in a decrease in fluorescence emission efficiency [57,58]. Therefore, the higher PEI content led to the observed lower fluorescence intensity. Additionally, with an increase in CA content, there are more carbon sources available that facilitate improved carbonization and the development of sp^2^-hybridized aromatic regions, which serve as the primary centers for light emission, thereby leading to enhanced fluorescence intensities [59,60].

### 3.2. Sensor Applications of BPEI CDs to Metal Ions

Carbon dots (CDs) represent a category of nanomaterials that have attracted considerable interest due to their prospective uses in detecting metal ions, attributed to their distinctive optical characteristics and biocompatibility [1,2,3,4]. The operational mechanism of carbon dots as sensors is fundamentally based on their capacity to engage with metal ions via multiple binding sites present on their surface, resulting in alterations to their photoluminescence properties [6,22,52]. Upon the binding of metal ions to the carbon dots, the fluorescence may either be quenched or enhanced, contingent upon the specific ion involved and the nature of the interaction [3,61,62]. This responsiveness to metal ions renders carbon dots an essential instrument for environmental surveillance, biomedical uses, and food safety [63,64,65]. The significance of these sensor applications is not solely rooted in their exceptional sensitivity and selectivity but also in their capability for real-time monitoring and provision of swift results, which are vital for prompt decision-making across various domains.

**Figure 3:**
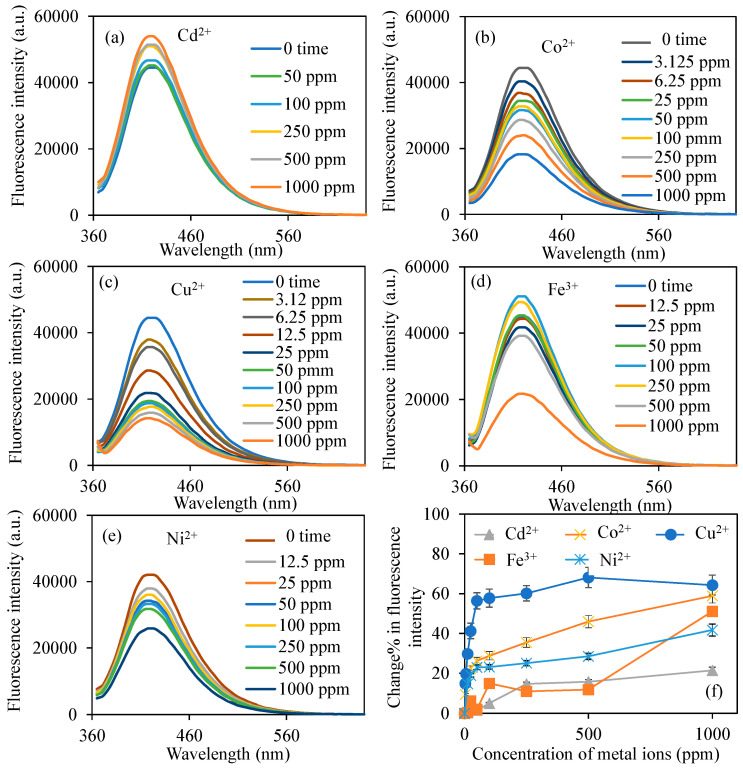
The fluorescence spectra of the B-PEI^1^ CDs in the presence of different concentrations of (**a**) Cd^2+^, (**b**) Co^2+^, (**c**) Cu^2+^, (**d**) Fe^3+^, (**e**) Ni^2+^ ions, and (**f**) change% in fluorescence intensity of BPEI^1^ CDs in the presence of related metal ions. [Excitation wavelength for BPEI CDs: 360 nm; emission wavelengths: 420 nm; the volume of metal ion solutions is 2 mL, and final concentration of BPEI CDs is 0.6 mg/mL].

Considering this information, an investigation was conducted into the alterations in the fluorescence characteristics of BPEI CDs synthesized in the presence of various metal ions. Figure 3 illustrates the modifications in the fluorescence properties of BPEI^1^ CDs in solutions formulated from the chloride salts of different metal ions, including Cd^2+^, Co^2+^, Cu^2+^, Fe^3+^, and Ni^2+^. The measurements were performed within the wavelength range of 360–640 nm, utilizing a light source at a PMT voltage of 600 V and an excitation wavelength of 360 nm. Figure 3a depicts the fluorescence characteristics of BPEI^1^ CDs prepared at a concentration of 0.6 mg/mL in 2 mL solutions containing Cd^2+^ ions at concentrations ranging from 50 to 1000 ppm. During the measurements, it was noted that the fluorescence intensity of the BPEI^1^ CD solution rose from 44,500 to 54,000 a.u. as the concentration of Cd^2+^ ions increased to 1000 ppm. Figure 3b illustrates the variations in fluorescence intensity of BPEI^1^ CDs when exposed to Co^2+^ ions. Notably, even at a Co^2+^ concentration of 3.12 ppm in 2 mL, the fluorescence intensity of BPEI^1^ CD diminished from 44,500 to 40,300 a.u. Furthermore, as the concentration of Co^2+^ ions increased to 1000 ppm, the fluorescence intensity further declined to 18,300 a.u. On the other hand, Figure 3c illustrates the change in the fluorescence characteristics of BPEI^1^ CDs when placed in solutions with differing concentrations of Cu^2+^ ions. Consequently, a reduction in the fluorescence properties of BPEI^1^ CDs was noted in relation to the concentration of Cu^2+^ ions. The fluorescence intensity of BPEI^1^ CDs, initially measured at 44,500 a.u., diminished to 37,900 a.u. with the introduction of just 2 mL of 3.12 ppm Cu^2+^ ions, and further declined to 15,850 a.u. as the concentration of Cu^2+^ ions escalated to 1000 ppm.

The change in the fluorescence characteristics of BPEI^1^ carbon dots (CDs) when exposed to Fe^3+^ and Ni^2+^ ions, which were utilized as metal ion species, were also examined, and the corresponding graphs are presented in Figure 3d and Figure 3e, respectively. Consequently, it was observed that the fluorescence properties of BPEI^1^ CDs varied in the presence of various concentrations of Fe^3+^ ion solution. While a decrease in fluorescence intensity was observed at high concentrations (>500 ppm), an increase in fluorescence was observed at relatively low concentrations (100–250 ppm). In the presence of 2 mL of 50 ppm Fe^3+^ ion solution, the fluorescence intensity value decreased from 44,500 a.u. to 41,760 a.u. On the other hand, no notable alteration was detected in the fluorescence characteristics of BPEI^1^ CDs when subjected to 2 mL of less than 12.5 ppm concentration of Ni^2+^ ion solutions. The prominent change noted was a reduction in the fluorescence intensity of BPEI^1^ CDs, which decreased from 44,500 to 38,000 a.u. in the presence of 2 mL of 12.5 ppm Ni^2+^ ion solution, and the fluorescence intensity further diminished to 25,900 with the increasing of Ni^2+^ ion concentration up to 1000 ppm.

In addition to these metal ions, the change in the fluorescence properties of BPEI^1^ CDs were also investigated in the presence of Ba^2+^, Ca^2+^, Mg^2+^, Mn^2+^, and Pb^2+^ ions. Since no significant change was observed in the fluorescence properties of BPEI^1^ CDs in the sensor studies performed with these metal ions, the graphs of the studies are given in Figure S1a–e. In brief, the fluorescence intensity value of BPEI^1^ CDs decreased to 41,620, and 44,410 a.u. in the presence of 2 mL of 1000 ppm Ba^2+^, and Mn^2+^ ion solutions, respectively, whereas it increased to 47,760, 46,230, and 48,000 a.u. in the presence of 2 mL of 1000 ppm Ca^2+^, Mg^2+^, and Pb^2+^ ion concentration, respectively.

Moreover, the highest change in fluorescence intensity in the presence of Co^2+^ and Cu^2+^ ions for the BPEI^1^ CDs against metal ions with various concentrations up to 100 ppm were performed and corresponding graphs were given in Figure S2a,b. The fluorescence intensity values of BPEI^1^ CDs decreased to 44,500 a.u. and 43,600 a.u. in the presence of 0.78 ppm of Co^2+^ ions, and 0.39 ppm of Cu^2+^ ions, respectively.

For a clearer understanding, the changes in the fluorescence characteristics of BPEI^1^ CDs, represented as a change% in fluorescence intensity against varying metal ion concentrations, are illustrated in Figure 3f. At lower metal ion concentrations, specifically at 3.12 ppm, the metal ions that exhibited variations in the fluorescence intensities of BPEI^1^ CDs were Co^2+^ and Cu^2+^, with the calculated changes in fluorescence intensities being 9.3 ± 0.05% and 14.8 ± 0.9%, respectively. Also, the change% in fluorescence intensity compared to Ba^2+^, Ca^2+^, Mg^2+^, Mn^2+^, and Pb^2+^ were also given in Figure S1f, and there is almost no change in the presence of related metal ions were observed.

On the other hand, as can be seen in Figure S2c, approximately 1.9% fluorescence intensity changes were observed even in the presence of 0.78 ppm of Co^2+^ ions, and 0.39 ppm of Cu^2+^ ions, respectively. Furthermore, as the concentrations of metal ions increased, the most significant change% values were noted for Co^2+^ and Cu^2+^ ions. Consequently, Figure S3a,b demonstrate a linear relationship between log(F_0_/F), where F_0_ represents the fluorescence intensity at time zero (prior to the addition of Co^2+^ and Cu^2+^ ions), F denotes the fluorescence intensity following the introduction of varying concentrations of Co^2+^ and Cu^2+^ ions in the solution, and the concentrations of the respective metal ions ranges from 0 to 50 ppm. This correlation can be expressed mathematically by the equation log(F_0_/F) = 0.0018x + 0.048, which has a correlation coefficient (R^2^) of 0.91 for Co^2+^, and log(F_0_/F) = 0.0061x + 0.0633, with a correlation coefficient (R^2^) of 0.99 for Cu^2+^ ions. Additionally, the limit of detection (LOD) for Co^2+^ and Cu^2+^ ions utilizing BPEI^1^ CDs was found to be 0.42 and 0.39 ppm, respectively, calculated from the equation, 3σ/slope where σ represents the standard deviation. Standard deviations were easily obtained from a regression analysis of the calibration curve by using data analysis add-ins in Excel. The calculated standard deviation values were 0.000411 for the Co^2+^ ion and 0.0007291 for the Cu^2+^ ion calibration curves, respectively.

### 3.3. Sensor Applications of BPEI CDs to Cu^2+^ Ions

**Figure 4:**
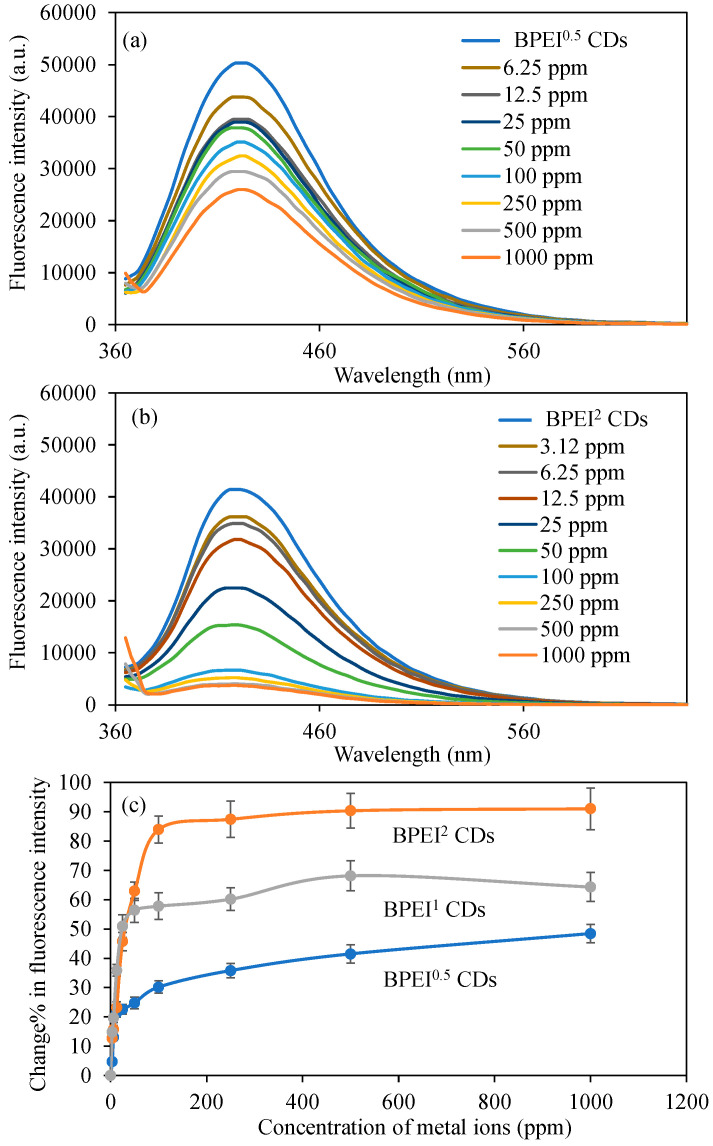
The fluorescence spectra of the (**a**) B-PEI^0.5^ and (**b**) B-PEI^2^ CDs in the presence of different concentrations of Cu^2+^ ions, and (**c**) change% in fluorescence intensity of BPEI CDs in the presence of different concentrations of Cu^2+^ ions. [Excitation wavelength for BPEI CDs: 360 nm; emission wavelengths: 420 nm; the volume of metal ion solutions is 2 mL, and final concentration of BPEI CDs is 0.6 mg/mL].

Given the reduced LOD value and increased fluorescence change% in comparison to other metal ions, our research proceeded with more comprehensive investigations involving the Cu^2+^ ion. For these investigations focused on the Cu^2+^ ion, the initial step involved examining the variations in fluorescence intensity values of BPEI^0.5^ and BPEI^2^ carbon dots (CDs) that contained varying proportions of polyethyleneimine (PEI) in solutions with different concentrations of Cu^2+^ ions. The corresponding graphs illustrating these findings are presented in Figure 4a,b. The fluorescence intensity of 0.6 mg/mL BPEI^0.5^ CDs in an aqueous solution was recorded at 50,300 a.u. However, this fluorescence intensity diminished to 43,800 a.u. in the presence of 2 mL 6.25 ppm Cu^2+^ ions. As the concentration of Cu^2+^ ions escalated, a corresponding further reduction in fluorescence intensity was observed, ultimately dropping to 26,000 a.u. in 2 mL of solution containing 1000 ppm Cu^2+^ ions. Furthermore, the fluorescence intensity of BPEI^2^ CDs, which possess a higher ratio of PEI, decreased from 41,400 to 36,100 a.u. when exposed to 2 mL of 3.12 ppm Cu^2+^ ion solution. Similarly, the reduction in fluorescence intensity was amplified with rising concentrations of Cu^2+^ ions. In a 2 mL solution with a concentration of 1000 ppm Cu^2+^ ions, the fluorescence intensity of BPEI^2^ CDs plummeted to 3700 a.u.

The changes in the fluorescence characteristics of BPEI^0.5^ and BPEI^2^ CDs were quantified as the change% in fluorescence intensity relative to different concentrations of Cu^2+^ metal ions, as illustrated in Figure 4c. Notably, a significant change% of 13.0 ± 0.9% in fluorescence intensity was recorded for BPEI^0.5^ CDs exclusively at a Cu^2+^ ion concentration of 6.25 ppm, whereas BPEI^2^ exhibited a notable decrease on fluorescence intensity of 12.7 ± 1.1% at a concentration of 3.12 ppm Cu^2+^ ions. Furthermore, for BPEI^1^ CDs, the calculated value was 14.8 ± 0.9% at the same concentration of 3.12 ppm Cu^2+^ ions, as previously depicted in Figure 3f, and included in Figure 4c for full comparison. Moreover, for BPEI^0.5^ and BPEI^2^ CDs, the fluorescence variations in the presence of different concentrations of Cu^2+^ ions were graphed as F0/F versus concentration, with the linear segments illustrated in Figure S4a,b. This relationship can be mathematically represented by the equation log(F_0_/F) = 0.004x + 0.0669 with an R^2^ value of 0.84 for BPEI^0.5^, and log(F_0_/F) = 0.0077x + 0.0385 with an R^2^ value of 0.99 for BPEI^2^ ions. Furthermore, the limit of detection (LOD) values for Cu^2+^ ions using BPEI^0.5^ and BPEI^2^ CDs were determined to be 0.53 and 0.30 ppm, respectively, calculated using the formula 3σ/slope. The observed phenomena can be elucidated by the varying number of nitrogen functionalities (–NH_2_, –CONH–, –C–N–, etc.) present on the surface of the BPEI CDs. It is important to note that the affinity for Cu^2+^ ions arises from the amine functionalities associated with BPEI-based CDs, thus the anticipated increase in response to Cu^2+^ ions corresponding to higher PEI content is a reasonable outcome.

**Figure 5:**
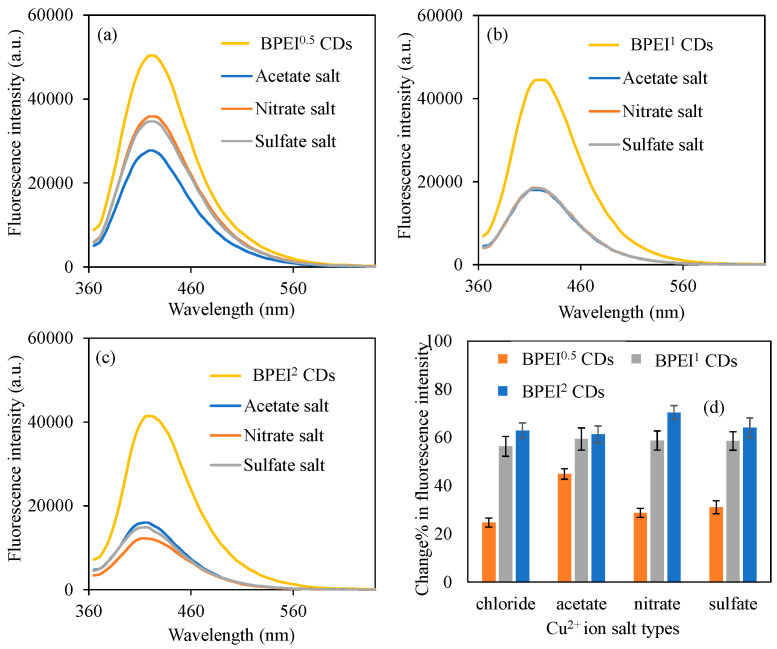
The fluorescence spectra of the (**a**) B-PEI^0.5^, (**b**) B-PEI^1^, and (**c**) B-PEI^2^ CDs in the presence of 2 mL 50 ppm concentrations of Cu^2+^ ion solution prepared from different salts, and (**d**) corresponding change% in fluorescence intensity of the BPEI CDs in the presence of those same Cu^2+^ ion salt solutions. [Excitation wavelength for BPEI CDs: 360 nm; emission wavelengths: 420 nm; the volume of metal ion solutions is 2 mL, and final concentration of BPEI CDs is 0.6 mg/mL].

In order to observe the effect of metal salts on the quenching ability of BPEI CDs, different salts of Cu^2+^ ions such as acetate, nitrate, and sulfate at 50 ppm concentrations, were also investigated. For this purpose, 0.6 mg/mL concentrations of BPEI CDs solution were prepared in 2 mL of 50 ppm Cu^2+^ solution, which were prepared from the acetate, nitrate, and sulfate salts of copper. The findings are illustrated in Figure 5. In Figure 5a, it is observed that the fluorescence intensity of BPEI^0.5^ CDs diminished from 50,300 a.u. to 27,800, 35,900, and 34,700 a.u. when exposed to 2 mL of 50 ppm Cu^2+^ ions of different anions, e.g., acetate, nitrate, and sulfate anions, respectively. The fluorescence characteristics of BPEI^0.5^ CDs are particularly notable, as they exhibit significant variations depending on the solutions derived from different salts of Cu^2+^ ions. Conversely, as illustrated in Figure 5b, the fluorescence intensity of BPEI^1^ CDs, which were synthesized from acetate, nitrate, and sulfate salts, displayed nearly identical fluorescence values at a concentration of 2 mL of 50 ppm Cu^2+^ ions. Specifically, these values were recorded as fluorescence intensities of 18,000, 18,300, and 18,400 a.u. for the Cu^2+^ ion solutions sourced from acetate, nitrate, and sulfate salts, respectively.

Furthermore, Figure 5c presents the fluorescence values of BPEI^2^ CDs, which are characterized by a higher concentration of nitrogen functionalization groups, highlighting the changes observed in 2 mL of 50 ppm solutions derived from various salts of Cu^2+^ ions. The study indicates that the fluorescence intensity values of BPEI^2^ CDs in the presence of Cu^2+^ ion solutions prepared from acetate, nitrate, and sulfate salts decreased to 16,000, 12,300, and 14,900 a.u., respectively. Additionally, the fluorescence intensity values of BPEI^0.5^, BPEI^1^, and BPEI^2^ CDs in 2 mL of 50 ppm Cu^2+^ ion solutions prepared from chloride salts experienced a decline from 50,300, 44,500, and 41,400 a.u. to 37,900, 19,400, and 15,400 a.u., respectively. The observed variations in quenching values among Cu^2+^ ion solutions prepared from these different salts can be attributed to both the ionic radius values of the corresponding anions and the surface functionalization present in the respective BPEI CDs. In Figure 5d, to facilitate a relative comparison, the change% in fluorescence intensity values of BPEI CDs in 2 mL of 50 ppm Cu^2+^ ion solutions derived from various salts are presented. Consequently, the fluorescence intensity values for BPEI^0.5^, BPEI^1^, and BPEI^2^ CDs in Cu^2+^ ion solutions sourced from chloride salt were determined to be 24.7 ± 1.8, 56.4 ± 4.1, and 62.9 ± 3.1%, respectively. The change in fluorescence intensity values of BPEI CDs in Cu^2+^ ion solutions formulated from acetate salt were calculated as 44.9 ± 2.2, 59.4 ± 4.6, and 61.3 ± 3.6% for BPEI^0.5^, BPEI^1^, and BPEI^2^ CDs, respectively. The change% in fluorescence intensity values for BPEI^0.5^, BPEI^1^, and BPEI^2^ CDs in the context of Cu^2+^ ion solutions prepared from nitrate salt were assessed as 28.7 ± 1.8, 58.7 ± 3.9, and 70.3 ± 2.9%. Lastly, the fluorescence intensity values of BPEI^0.5^, BPEI^1^, and BPEI^2^ CDs exhibited changes of 31.1 ± 2.7, 58.6 ± 3.9, and 64.1 ± 4.1% in the presence of Cu^2+^ ion solutions derived from sulfate salts, respectively. The increased change% in the fluorescence intensities of BPEI^2^ CDs, in comparison to other BPEI CDs across all Cu^2+^ ion solutions prepared from different salts, was attributed to their nitrogen-based functional groups. Furthermore, the most significant change% in fluorescence intensity for BPEI^0.5^ CDs was noted in Cu^2+^ ion solutions prepared from acetate salt, while for BPEI^2^ CDs, it was observed in Cu^2+^ ion solutions prepared from nitrate salt, which was ascribed to the presence of analogous atomic functional groups. Additionally, the possibility of more carboxylic acid groups on BPEI^0.5^ CDs due to low BPEI ratio and more functional groups containing nitrogen groups on BPEI^2^ CDs due to high PEI ratio can be the main reason for the variation in the reduction in the fluorescence intensities of the different BPEI CDs.

### 3.4. Selectivity of BPEI CDs Sensors to Cu^2+^ Ions

**Figure 6:**
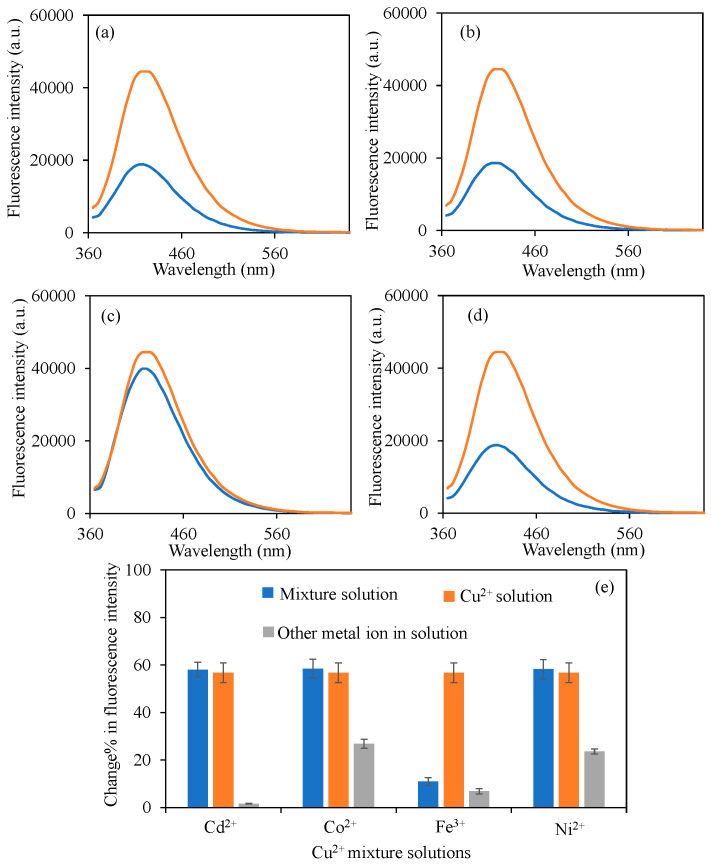
The fluorescence spectra of the B-PEI^1^ CDs in the presence of 2 mL 50 ppm concentrations of (**a**) Cu^2+^-Cd^2+^, (**b**) Cu^2+^-Co^2+^, (**c**) Cu^2+^-Fe^3+^, (**d**) Cu^2+^-Ni^2+^ mixture solutions, and (**e**) change% in fluorescence intensity of BPEI^1^ CDs in the presence of 2 mL 50 ppm mixture solutions. [Excitation wavelength for BPEI CDs: 360 nm; emission wavelengths: 420 nm; the volume of metal ion solutions is 2 mL, and final concentration of BPEI CDs is 0.6 mg/mL. In (**a**–**d**), orange-lined spectrum corresponds to bare BPEI^1^ CDs in water, blue line in the spectrum is BPEI^1^ CDs in mixture solutions].

In the literature, many carbon dots with various heteroatom doping were reported as potential sensors for different metal ions such as Cu^2+^, Fe^3+^, Hg^2+^, Cr^3+^, Al^3+^, etc. [66,67,68,69,70,71]. Some of the reported studies with LOD values were summarized in Table S1. As can clearly be seen from the table that the types of the heteroatoms used for doping, the precursors, synthesis method, and even synthesis temperature and carbon dot sizes are very important factors that affect the sensitivity and selectivity of the prepared carbon dots for various metal ions. Selectivity of sensors is one of the most important properties in their application, in addition to their sensitivity. Here, the sensitivity of BPEI^1^ CDs with 0.39 ppm to Cu^2+^ ions has a competitive result with the results found in the literature [72,73,74,75]. In drinking water, the permissible concentration of Cu^2+^ has been established at 2 ppm by the WHO, and 1.3 ppm (20 μM) by the EPA [31,32]. Consequently, the BPEI^1^ CDs fluorescence intensity changes can be effectively utilized for the identification of Cu^2+^. Furthermore, the selectivity towards Cu^2+^ ions was examined in the context of other metal ions, specifically Cd^2+^, Co^2+^, Ni^2+^, and Pb^2+^, both individually and in a mixture of all metal ions, with the corresponding graphs illustrated in Figure 6. All metal ion solutions were derived from their respective chloride salts. The fluorescence intensity of BPEI^1^ carbon dots (CDs) was measured at 44,500 a.u. As depicted in Figure 6a, the fluorescence intensity of BPEI^1^ CDs diminished to 18,800 a.u. when subjected to a 2 mL solution of a 50 ppm Cu^2+^-Cd^2+^ mixture. Comparatively, Figure 6b indicates that the fluorescence intensity in a 2 mL solution of a 50 ppm Cu^2+^-Co^2+^ mixture decreased to 18,600 a.u. Different from the other metal ions, in Figure 6c, in the presence of 2 mL solution of a 50 ppm Cu^2+^-Fe^3+^ mixture, the fluorescence intensity decreased to 39,940 a.u., which shows Fe^3+^ ions interference during Cu^2+^ ion sensing and prevents further fluorescence intensity decrease. This was explained in a similar study with the N/O ratio on the surface of CDs [76]. Also, it was reported that Fe^3+^ strongly competes (or even dominates) the quenching [18,77]. Therefore, to eliminate this interference surface functionalities of BPEI CDs need to be adjusted carefully.

The alteration in fluorescence intensity values of BPEI^1^ CDs in a 2 mL solution of a 50 ppm Cu^2+^-Ni^2+^ mixture is presented in Figure 6d, where the fluorescence value was noted to decline to 18,700 a.u. On the other hand, the other metal ions mentioned above were also used to prepare Cu^2+^ ion mixtures, and the quenching ability of BPEI^1^ CDs in the presence of 2 mL of 50 ppm Cu^2+^-Ba^2+^, Cu^2+^-Ca^2+^, Cu^2+^-Mg^2+^, Cu^2+^-Mn^2+^, and Cu^2+^-Pb^2+^ mixtures were also investigated, and the corresponding fluorescence spectra were given inFigure S5a–e, respectively. The fluorescence spectrum of BPEI^1^ CDs in the presence of related metal ion mixtures is almost similar to the fluorescence spectrum of a bare 50 ppm Cu^2+^ ion solution. Moreover, the change% in fluorescence intensity in solutions containing solely Cu^2+^ and other metal ions, along with the change% of Cu^2+^ ions in both individual and collective mixtures with other metal ions, are also compared as illustrated in Figure 6e and Figure S5f. The results indicate that the change% in fluorescence intensity across all mixtures with the presence of Cu^2+^ ions alone remained relatively consistent except in the presence of Fe^3+^ ions, thereby demonstrating the selectivity of the synthesized BPEI^1^ CDs for Cu^2+^ ions.

**Figure 7:**
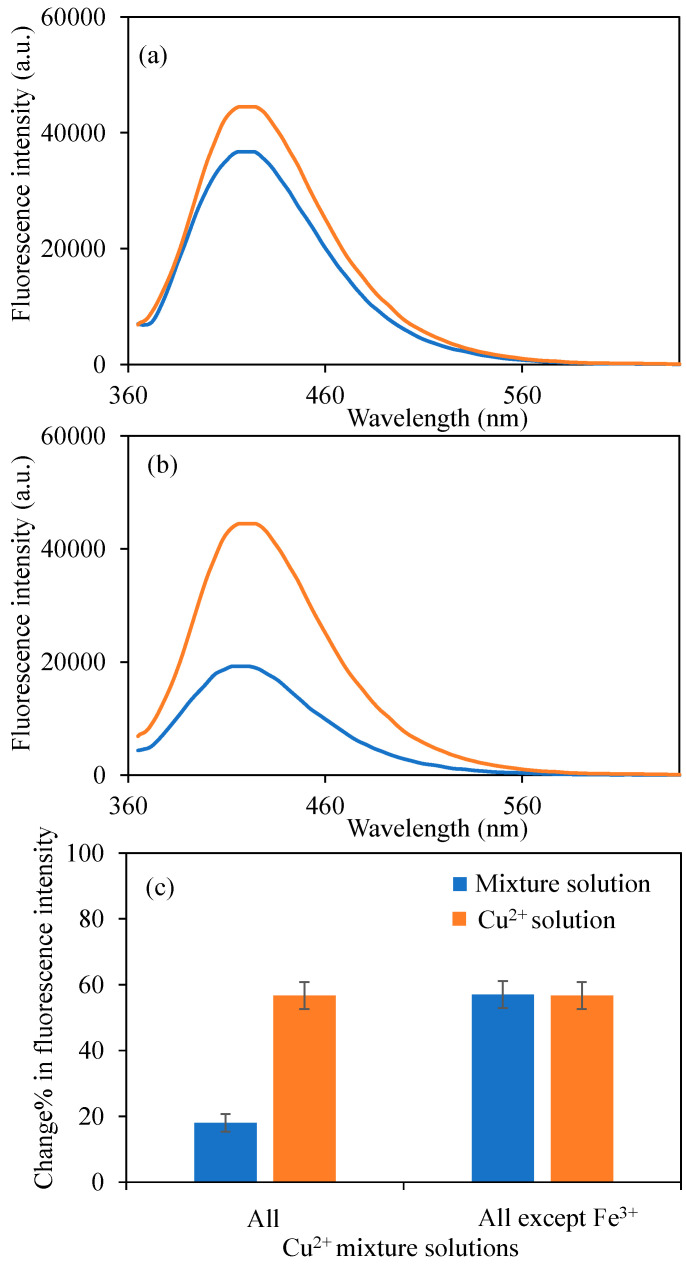
The fluorescence spectra of the B-PEI^1^ CDs in the presence of 2 mL 50 ppm concentrations of (**a**) all used metal ions, and (**b**) all used metal ions except Fe^3+^ mixture solutions, and (**c**) change% in fluorescence intensity of BPEI^1^ CDs in the presence of 2 mL 50 ppm mixture solutions. [Excitation wavelength for BPEI CDs: 360 nm; emission wavelengths: 420 nm; the volume of metal ion solutions is 2 mL, and final concentration of BPEI CDs is 0.6 mg/mL. Blue-lined spectrum corresponds to bare BPEI^1^ CDs in different waters, orange line in the spectrum corresponds to BPEI^1^ CDs in Cu^2+^ ion contained different waters].

Moreover, the fluorescence intensity of BPEI^1^ CDs in the presence of all metal ions mixture solutions and except for Fe^3+^ metal ions, at 50 ppm for each metal ion, and a total volume of 2 mL were also investigated, and corresponding graphs were presented in Figure 7a and Figure 7b, respectively. It was clearly seen from Figure 7a that, even in 10 metal ions containing mixture solutions, the presence of 50 ppm Fe^3+^ metal ions affected the sensing ability of Cu^2+^ ions by BPEI^1^ CDs.

On the other hand, in the presence of 9 used metal ions except Fe^3+^ ions, in Figure 7b, the fluorescence intensity of BPEI^1^ CDs solution at 0.6 mg/mL concentrations decreased to 19,280 a.u. The change% in fluorescence intensity across all metal ions containing mixtures, and the Fe^3+^ ions exception one was also given in Figure 7c. From the selectivity results, the presence of equal concentrations of Fe^3+^ ions with Cu^2+^ ions (50 ppm), the selective sensing ability of BPEI^1^ CDs to Cu^2+^ ions was negatively affected. However, this problem can be solved by adjusting the surface functionalities of BPEI^1^ CDs.

### 3.5. Cu^2+^ Ion Detections in Real Water Samples

**Figure 8:**
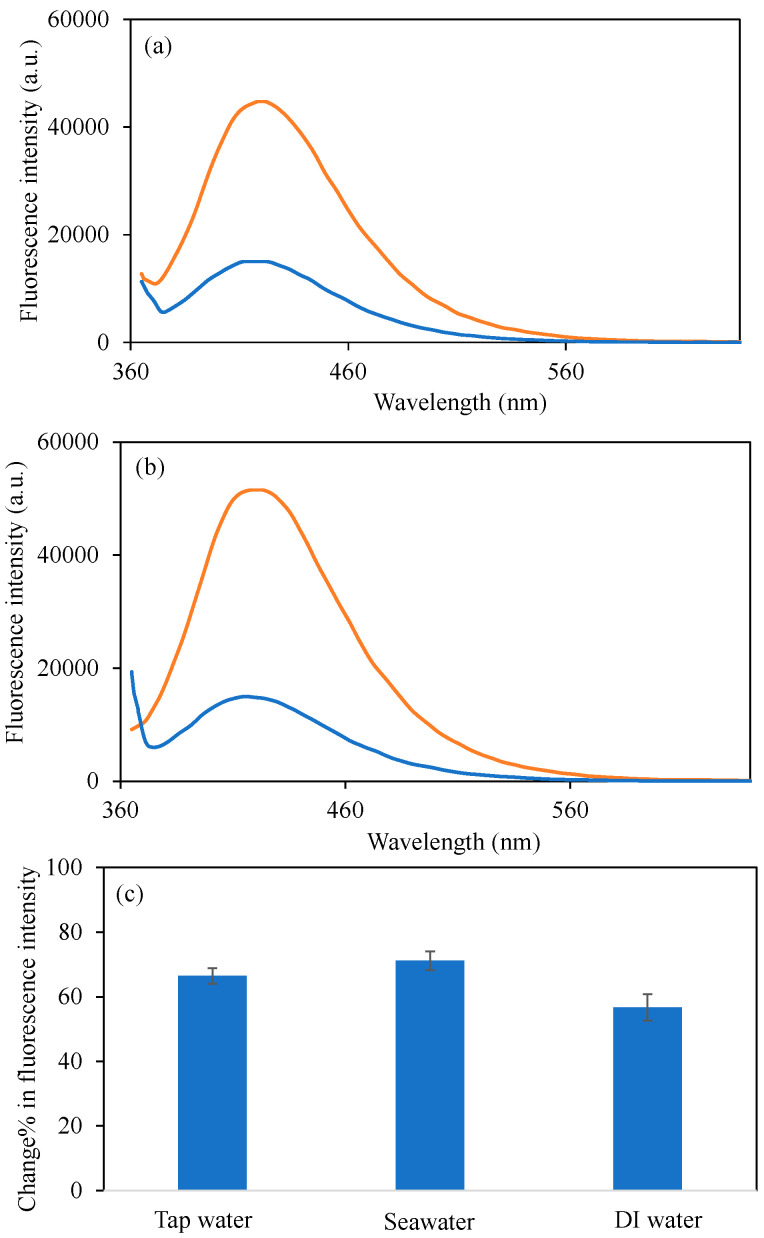
The fluorescence spectra of the B-PEI^1^ CDs in the presence of 2 mL 50 ppm concentrations of Cu^2+^ion solutions (**a**) in tap water, (**b**) in seawater, and (**c**) change% in fluorescence intensity of BPEI^1^ CDs in the presence of 2 mL 50 Cu^2+^ion solutions in different waters. [Excitation wavelength for BPEI CDs: 360 nm; emission wavelengths: 420 nm; the volume of metal ion solutions is 2 mL, and final concentration of BPEI CDs is 0.6 mg/mL. The blue-lined spectrum corresponds to bare BPEI^1^ CDs in different waters, whereas the orange line corresponds to BPEI^1^ CDs in Cu^2+^ ion contained different waters].

The aforementioned studies have effectively illustrated that the synthesized BPEI^1^ CDs exhibit commendable sensor capabilities in detecting Cu^2+^ ions in distilled water. Nevertheless, given the existence of a variety of anions, cations, salts, minerals, and even microbial entities in actual water samples such as tap water and seawater, it is imperative for the sensors to maintain equivalent or comparable performance in these real water conditions. Consequently, the variations in fluorescence intensity of the synthesized BPEI^1^ CDs in 50 ppm Cu^2+^ solutions prepared in both tap and seawater were also examined, with the pertinent graphs presented in Figure 8. In Figure 8a, the fluorescence intensity of BPEI^1^ CDs at a concentration of 0.6 mg/mL was recorded at 44,700 a.u. in tap water, which diminished to 15,000 a.u. upon the introduction of a 50 ppm Cu^2+^ ion solution in tap water.

Furthermore, the alterations in fluorescence intensity in seawater with the presence of 50 ppm Cu^2+^ ions are depicted in Figure 8b. Accordingly, the fluorescence intensity of BPEI^1^ CDs, which initially registered a value of 51,500 a.u. at a concentration of 0.6 mg/mL in seawater, decreased to 14,800 a.u. in seawater containing 50 ppm Cu^2+^. The change% in fluorescence intensity values for 50 ppm Cu^2+^ ion solutions in tap water, seawater, and distilled water are illustrated in Figure 8c and were calculated to be 66.4 ± 2.4%, 71.2 ± 2.9%, and 56.7 ± 4.1%, respectively. In conclusion, it has been demonstrated that BPEI^1^ CDs can be effectively utilized as sensors for Cu^2+^ ions in real water environments.

## 4. Conclusions

In conclusion, our earlier research has indicated that BPEI CD structures, which have been thoroughly characterized, have been shown to possess important qualities for biomedical use. Here, we show that they can also serve as sensitive and selective sensors for Cu^2+^ ions. In this study, the lowest detection (LOD) value of Cu^2+^ ions recorded with the use of BPEI^2^ CDs is linked to its higher number of nitrogen-derived structures in its composition. BPEI-based CDs tested in both tap water and seawater, exhibit a high fluorescence quenching ability comparable to that of distilled water, making them suitable for use as Cu^2+^ sensors in natural water sources. Given that Cu^2+^ ions can enter the human body through environmental waters and that excessive accumulation of Cu^2+^ can result in various health issues, the BPEI CD structures developed in this research are believed to hold promise for water testing directly related to diagnostic applications. Nevertheless, it is crucial to emphasize that further detailed investigations are still required.

## Data Availability

No new data used during the study and the generated data is used within this study.

## Supplementary Materials

The following supporting information can be downloaded at https://www.mdpi.com/article/10.3390/mi16111275/s1: Table S1. Comparisons of some of the reported studies on the potential sensor applications of CDs for different metal ions. Figure S1: The linear relationship between the log(F_0_/F) and the Cu^2+^ concentration, where F and F_0_ stand for the concentrations of Cu^2+^ ions in the presence of BPEI^1^ CDs. Figure S2: The linear relationship between the log(F_0_/F) and the Cu^2+^ concentration, where F and F_0_ stand for the concentrations of Cu^2+^ ions in the presence of (**a**) BPEI^0.5^, and (**b**) BPEI^2^ CDs. Figure S3: The linear relationship between the log(F_0_/F) and the (**a**) Co^2+^, (**b**) Cu^2+^concentration, where F and F_0_ stand for the concentrations of Cu^2+^ ions in the presence of BPEI^1^ CDs. Figure S4: The linear relationship between the log(F_0_/F) and the Cu^2+^ concentration, where F and F_0_ stand for the concentrations of Cu^2+^ ions in the presence of (**a**) BPEI^0.5^, and (**b**) BPEI^2^ CDs. Figure S5. The fluorescence spectra of the B-PEI^1^ CDs in the presence of 2 mL 50 ppm concentrations of (**a**) Cu^2+^-Ba^2+^, (**b**) Cu^2+^-Ca^2+^, (**c**) Cu^2+^-Mg^2+^, (**d**) Cu^2+^-Mn^2+^, (**e**) Cu^2+^-Pb^2+^ mixture solution, and (**f**) change% in fluorescence intensity of BPEI^1^ CDs in the presence of 2 mL 50 ppm mixture solutions. [Excitation wavelength for BPEI CDs: 360 nm; emission wavelengths: 420 nm; the volume of metal ion solutions is 2 mL, and the final concentration of BPEI CDs is 0.6 mg/mL. Orange-lined spectrum is bare BPEI^1^ CDs in water, blue-lined spectrum is BPEI^1^ CDs in mixture solutions.]

## References

[1] You W., Zou W., Jiang S., Zhang J., Ge Y., Lu G., Bahnemann D.W., Pan J.H. (2024). Fluorescent Carbon Quantum Dots with Controllable Physicochemical Properties Fantastic for Emerging Applications: A Review. Carbon Neutralization.

[2] Sahana S., Gautam A., Singh R., Chandel S. (2023). A Recent Update on Development, Synthesis Methods, Properties and Application of Natural Products Derived Carbon Dots. Nat. Prod. Bioprospect..

[3] Qureshi Z.A., Dabash H., Ponnamma D., Abbas M.K.G. (2024). Carbon Dots as Versatile Nanomaterials in Sensing and Imaging: Efficiency and Beyond. Heliyon.

[4] Xu J., Huang B.-B., Lai C.-M., Lu Y.-S., Shao J.-W. (2024). Advancements in the Synthesis of Carbon Dots and Their Application in Biomedicine. J. Photochem. Photobiol. B Biol..

[5] Zhang P., Zheng Y., Ren L., Li S., Feng M., Zhang Q., Qi R., Qin Z., Zhang J., Jiang L. (2024). The Enhanced Photoluminescence Properties of Carbon Dots Derived from Glucose: The Effect of Natural Oxidation. Nanomaterials.

[6] Lou X.-T., Zhan L., Chen B.-B. (2025). Recent Progress of Carbon Dots in Fluorescence Sensing. Inorganics.

[7] Zhu S., Song Y., Zhao X., Shao J., Zhang J., Yang B. (2015). The Photoluminescence Mechanism in Carbon Dots (Graphene Quantum Dots, Carbon Nanodots, and Polymer Dots): Current State and Future Perspective. Nano Res..

[8] Gao K., Sun S., Zhang B. (2024). Recent Advancements in the Development of Graphene-Based Materials for Catalytic Applications. ChemCatChem.

[9] Abbas Q., Raza R., Shabbir I., Olabi A.G. (2019). Heteroatom Doped High Porosity Carbon Nanomaterials as Electrodes for Energy Storage in Electrochemical Capacitors: A Review. J. Sci. Adv. Mater. Devices.

[10] Munusamy S., Mandlimath T.R., Swetha P., Al-Sehemi A.G., Pannipara M., Koppala S., Shanmugam P., Boonyuen S., Pothu R., Boddula R. (2023). Nitrogen-Doped Carbon Dots: Recent Developments in Its Fluorescent Sensor Applications. Environ. Res..

[11] Basha D.B., Ahmed S., Ahmed A., Gondal M.A. (2023). Recent Advances on Nitrogen Doped Porous Carbon Micro-Supercapacitors: New Directions for Wearable Electronics. J. Energy Storage.

[12] Nguyen K.G., Baragau I.-A., Gromicova R., Nicolaev A., Thomson S.A.J., Rennie A., Power N.P., Sajjad M.T., Kellici S. (2022). Investigating the Effect of N-Doping on Carbon Quantum Dots Structure, Optical Properties and Metal Ion Screening. Sci. Rep..

[13] Park Y., Kim Y., Chang H., Won S., Kim H., Kwon W. (2020). Biocompatible Nitrogen-Doped Carbon Dots: Synthesis, Characterization, and Application. J. Mater. Chem. B.

[14] Qu D., Zheng M., Li J., Xie Z., Sun Z. (2015). Tailoring Color Emissions from N-Doped Graphene Quantum Dots for Bioimaging Applications. Light Sci. Appl..

[15] Xu M., He G., Li Z., He F., Gao F., Su Y., Zhang L., Yang Z., Zhang Y. (2014). A Green Heterogeneous Synthesis of N-Doped Carbon Dots and Their Photoluminescence Applications in Solid and Aqueous States. Nanoscale.

[16] Shabbir H., Csapó E., Wojnicki M. (2023). Carbon Quantum Dots: The Role of Surface Functional Groups and Proposed Mechanisms for Metal Ion Sensing. Inorganics.

[17] Xu X., Li Y., Hu G., Mo L., Zheng M., Lei B., Zhang X., Hu C., Zhuang J., Liu Y. (2020). Surface Functional Carbon Dots: Chemical Engineering Applications beyond Optical Properties. J. Mater. Chem. C Mater..

[18] Hasan M., Baheerathan B., Sutradhar S., Shahbandinejad R., Rakshit S., Kozinski J., Li D., Hu Y., Kang K. (2025). Microwave-Assisted Synthesis of Biomass-Derived N-Doped Carbon Dots for Metal Ion Sensing. Carbon Res..

[19] Yuan F., Li S., Fan Z., Meng X., Fan L., Yang S. (2016). Shining Carbon Dots: Synthesis and Biomedical and Optoelectronic Applications. Nano Today.

[20] Zhang Z., Wang Y., Guo T., Hu P. (2025). The Influence of Defect Engineering on the Electronic Structure of Active Centers on the Catalyst Surface. Catalysts.

[21] Ren Y., Geng W., Xu R., Wang P., Zhao H. (2025). Tuning Electronic and Pore Structures of Biochar via Nitrogen and Magnesium Doping for Superior Methylene Blue Adsorption: Synergistic Mechanisms and Kinetic Analysis. ACS Omega.

[22] Lamba R., Yukta Y., Mondal J., Kumar R., Pani B., Singh B. (2024). Carbon Dots: Synthesis, Characterizations, and Recent Advancements in Biomedical, Optoelectronics, Sensing, and Catalysis Applications. ACS Appl. Bio Mater..

[23] Sudewi S., Sai Sashank P.V., Kamaraj R., Zulfajri M., Huang G.G. (2024). Understanding Antibiotic Detection with Fluorescence Quantum Dots: A Review. J. Fluoresc..

[24] Song Y., Xia X., Xiao Z., Zhao Y., Yan M., Li J., Li H., Liu X. (2022). Synthesis of N,S Co-Doped Carbon Dots for Fluorescence Turn-on Detection of Fe^2+^ and Al^3+^ in a Wide PH Range. J. Mol. Liq..

[25] Inagaki M., Toyoda M., Soneda Y., Morishita T. (2018). Nitrogen-Doped Carbon Materials. Carbon.

[26] Antolini E. (2016). Nitrogen-Doped Carbons by Sustainable N- and C-Containing Natural Resources as Nonprecious Catalysts and Catalyst Supports for Low Temperature Fuel Cells. Renew. Sustain. Energy Rev..

[27] Wu B., Meng H., Morales D.M., Zeng F., Zhu J., Wang B., Risch M., Xu Z.J., Petit T. (2022). Nitrogen-Rich Carbonaceous Materials for Advanced Oxygen Electrocatalysis: Synthesis, Characterization, and Activity of Nitrogen Sites. Adv. Funct. Mater..

[28] Ejaz A., Jeon S. (2018). The Individual Role of Pyrrolic, Pyridinic and Graphitic Nitrogen in the Growth Kinetics of Pd NPs on N-RGO Followed by a Comprehensive Study on ORR. Int. J. Hydrogen Energy.

[29] Filimon M.N., Caraba I.V., Popescu R., Dumitrescu G., Verdes D., Petculescu Ciochina L., Sinitean A. (2021). Potential Ecological and Human Health Risks of Heavy Metals in Soils in Selected Copper Mining Areas—A Case Study: The Bor Area. Int. J. Environ. Res. Public Health.

[30] Georgopoulos P.G., Roy A., Yonone-Lioy M.J., Opiekun R.E., Lioy P.J. (2001). Environmental Copper: Its Dynamics and Human Exposure Issues. J. Toxicol. Environ. Health Part B.

[31] World Health Organization (2024). Copper in Drinking-Water.

[32] The United States Environmental Protection Agency (2010). Lead and Copper Rule.

[33] Vo T.T.T., Peng T.-Y., Nguyen T.H., Bui T.N.H., Wang C.-S., Lee W.-J., Chen Y.-L., Wu Y.-C., Lee I.-T. (2024). The Crosstalk between Copper-Induced Oxidative Stress and Cuproptosis: A Novel Potential Anticancer Paradigm. Cell Commun. Signal..

[34] Zhang Z., Tang H., Du T., Yang D. (2024). The Impact of Copper on Bone Metabolism. J. Orthop. Transl..

[35] Sailer J., Nagel J., Akdogan B., Jauch A.T., Engler J., Knolle P.A., Zischka H. (2024). Deadly Excess Copper. Redox Biol..

[36] Gao L., Zhang A. (2023). Copper-Instigated Modulatory Cell Mortality Mechanisms and Progress in Oncological Treatment Investigations. Front. Immunol..

[37] de Bie P., van de Sluis B., Burstein E., van de Berghe P.V.E., Muller P., Berger R., Gitlin J.D., Wijmenga C., Klomp L.W.J. (2007). Distinct Wilson’s Disease Mutations in ATP7B Are Associated With Enhanced Binding to COMMD1 and Reduced Stability of ATP7B. Gastroenterology.

[38] Ovchinnikova E.V., Garbuz M.M., Ovchinnikova A.A., Kumeiko V.V. (2024). Epidemiology of Wilson’s Disease and Pathogenic Variants of the ATP7B Gene Leading to Diversified Protein Disfunctions. Int. J. Mol. Sci..

[39] Patil M., Sheth K.A., Krishnamurthy A.C., Devarbhavi H. (2013). A Review and Current Perspective on Wilson Disease. J. Clin. Exp. Hepatol..

[40] Liu M., Cohen E.J., Brewer G.J., Laibson P.R. (2002). Kayser-Fleischer Ring as the Presenting Sign of Wilson Disease. Am. J. Ophthalmol..

[41] Acıpayam C., Altunay A., İlhan N., Atçı N. (2015). Wilson’s Disease Presenting With Pancytopenia. Mustafa Kemal Üniversitesi Tıp Dergisi.

[42] Zhang D., Li Y., Pan J., Zheng Y., Xu X. (2024). Copper Homeostasis and Cuproptosis in Radiation-Induced Injury. Biomed. Pharmacother..

[43] Feng W., Su S., Song C., Yu F., Zhou J., Li J., Jia R., Xu P., Tang Y. (2022). Effects of Copper Exposure on Oxidative Stress, Apoptosis, Endoplasmic Reticulum Stress, Autophagy and Immune Response in Different Tissues of Chinese Mitten Crab (*Eriocheir sinensis*). Antioxidants.

[44] Pivariu D., Oros A.N., Tabaran A., Caloni F., Bolfa P., Nagy A.-L. (2024). Chronic Copper Bilysinate Poisoning in Five Texel Sheep: A Case Report. Life.

[45] Wang Y., Li D., Xu K., Wang G., Zhang F. (2025). Copper Homeostasis and Neurodegenerative Diseases. Neural Regen. Res..

[46] Li Y., Han Y., Shu Q., Kan Y.-K., Wang Z. (2025). Cuproptosis and Copper as Potential Mechanisms and Intervention Targets in Alzheimer’s Disease. Biomed. Pharmacother..

[47] Zhong G., Wang X., Li J., Xie Z., Wu Q., Chen J., Wang Y., Chen Z., Cao X., Li T. (2024). Insights Into the Role of Copper in Neurodegenerative Diseases and the Therapeutic Potential of Natural Compounds. Curr. Neuropharmacol..

[48] Twomey P.J., Viljoen A., House I.M., Reynolds T.M., Wierzbicki A.S. (2006). Adjusting Copper Concentrations for Caeruloplasmin Levels in Routine Clinical Practice. J. Clin. Pathol..

[49] Demirci S., McNally A.B., Ayyala R.S., Lawson L.B., Sahiner N. (2020). Synthesis and Characterization of Nitrogen-Doped Carbon Dots as Fluorescent Nanoprobes with Antimicrobial Properties and Skin Permeability. J. Drug Deliv. Sci. Technol..

[50] Demirci S., Suner S.S., Sahiner M., Akcali A., Guven O., Sahiner N. (2025). A Comparative Study of Nitrogen Doped Carbon Dots Prepared from Linear Polyethyleneimine (L-PEI) and Branched Polyethyleneimine (B-PEI): Thermal, Optical, Biocompatibility, Sensor, Antibacterial, and Light-Induced Antibacterial Activity. J. Fluoresc..

[51] Sousa H.B.A., Martins C.S.M., Prior J.A.V. (2021). You Don’t Learn That in School: An Updated Practical Guide to Carbon Quantum Dots. Nanomaterials.

[52] Liu M.L., Chen B.B., Li C.M., Huang C.Z. (2019). Carbon Dots: Synthesis, Formation Mechanism, Fluorescence Origin and Sensing Applications. Green Chem..

[53] Ma Z., Yu Y., Tao S., Han X., Zhang K., Yang B. (2025). Carbonized Polymer Dots: A Class of Highly Functionalized Nanoparticles with Polymeric Characteristics. Polym. Sci. Technol..

[54] Wang Z., Li F., Zhang L., Qian J., Cao S. (2020). Phase-Transfer-Assisted Synthesis of Cysteine-Ag Nanoparticles/Graphene Oxide Nanocomposite and Its Enhanced Performance in Antibiosis and Biosensing. Nanotechnology.

[55] Zhang Z., Yi G., Li P., Zhang X., Fan H., Zhang Y., Wang X., Zhang C. (2020). A Minireview on Doped Carbon Dots for Photocatalytic and Electrocatalytic Applications. Nanoscale.

[56] Hu R., Li L., Jin W.J. (2017). Controlling Speciation of Nitrogen in Nitrogen-Doped Carbon Dots by Ferric Ion Catalysis for Enhancing Fluorescence. Carbon.

[57] Li M., Wu Z., Ren W., Cheng H., Tang N., Wu W., Zhong W., Du Y. (2012). The Doping of Reduced Graphene Oxide with Nitrogen and Its Effect on the Quenching of the Material’s Photoluminescence. Carbon.

[58] Barman B.K., Okano K., Deguchi K., Ohki S., Hashi K., Goto A., Nagao T. (2022). N-Dopant Site Formulation for White-Light-Emitting Carbon Dots with Tunable Chromaticity. ACS Sustain. Chem. Eng..

[59] Osorio H.M., Castillo-Solís F., Barragán S.Y., Rodríguez-Pólit C., Gonzalez-Pastor R. (2024). Graphene Quantum Dots from Natural Carbon Sources for Drug and Gene Delivery in Cancer Treatment. Int. J. Mol. Sci..

[60] Liu J., Li R., Yang B. (2020). Carbon Dots: A New Type of Carbon-Based Nanomaterial with Wide Applications. ACS Cent. Sci..

[61] Qian Z., Ma J., Shan X., Feng H., Shao L., Chen J. (2014). Highly Luminescent N-Doped Carbon Quantum Dots as an Effective Multifunctional Fluorescence Sensing Platform. Chem.—Eur. J..

[62] Yi Z., Li X., Zhang H., Ji X., Sun W., Yu Y., Liu Y., Huang J., Sarshar Z., Sain M. (2021). High Quantum Yield Photoluminescent N-Doped Carbon Dots for Switch Sensing and Imaging. Talanta.

[63] Nguyen D.H.H., Muthu A., Elsakhawy T., Sheta M.H., Abdalla N., El-Ramady H., Prokisch J. (2025). Carbon Nanodots-Based Sensors: A Promising Tool for Detecting and Monitoring Toxic Compounds. Nanomaterials.

[64] Thanjavur N., Kim Y.-J. (2025). Illuminating Pollutants: The Role of Carbon Dots in Environmental Sensing. Chemosensors.

[65] Nguyen D.H.H., El-Ramady H., Prokisch J. (2025). Food Safety Aspects of Carbon Dots: A Review. Environ. Chem. Lett..

[66] Atchudan R., Perumal S., Edison T.N.J.I., Sundramoorthy A.K., Vinodh R., Sangaraju S., Kishore S.C., Lee Y.R. (2023). Natural Nitrogen-Doped Carbon Dots Obtained from Hydrothermal Carbonization of Chebulic Myrobalan and Their Sensing Ability toward Heavy Metal Ions. Sensors.

[67] Kalaiyarasan G., Joseph J., Kumar P. (2020). Phosphorus-Doped Carbon Quantum Dots as Fluorometric Probes for Iron Detection. ACS Omega.

[68] Zeng J., Liao L., Lin X., Liu G., Luo X., Luo M., Wu F. (2022). Red-Emissive Sulfur-Doped Carbon Dots for Selective and Sensitive Detection of Mercury (II) Ion and Glutathione. Int. J. Mol. Sci..

[69] Kamali S.R., Chen C.-N., Agrawal D.C., Wei T.-H. (2021). Sulfur-Doped Carbon Dots Synthesis under Microwave Irradiation as Turn-off Fluorescent Sensor for Cr(III). J. Anal. Sci. Technol..

[70] Chaghaghazardi M., Kashanian S., Nazari M., Omidfar K., Joseph Y., Rahimi P. (2023). Nitrogen and Sulfur Co-Doped Carbon Quantum Dots Fluorescence Quenching Assay for Detection of Mercury (II). Spectrochim. Acta A Mol. Biomol. Spectrosc..

[71] Mohandoss S., Ganesan S., Palanisamy S., You S., Velsankar K., Sudhahar S., Lo H.-M., Lee Y.R. (2023). Nitrogen, Sulfur, and Phosphorus Co-Doped Carbon Dots-Based Ratiometric Chemosensor for Highly Selective Sequential Detection of Al^3+^ and Fe^3+^ Ions in Logic Gate, Cell Imaging, and Real Sample Analysis. Chemosphere.

[72] Liu X., Zhang S., Xu H., Wang R., Dong L., Gao S., Tang B., Fang W., Hou F., Zhong L. (2020). Nitrogen-Doped Carbon Quantum Dots from Poly(Ethyleneimine) for Optical Dual-Mode Determination of Cu ^2+^ and l-Cysteine and Their Logic Gate Operation. ACS Appl. Mater. Interfaces.

[73] Chen Z., Han X., Lin Z., Fan Y., Shi G., Zhang S., Zhang M. (2019). Facile Reflux Synthesis of Polyethyleneimine-capped Fluorescent Carbon Dots for Sequential Bioassays toward Cu^2+^/H_2_S and Its Application for a Logic System. Biotechnol. Appl. Biochem..

[74] Hu G., Pei Z., Shen B., Li Y., Wei W., Zhang J., Li J. (2024). Correlation between Surface Structure of Carbon Dots and Selective Detection of Heavy Metal Ions. Appl. Phys. A.

[75] Yang J., Jin X., Cheng Z., Zhou H., Gao L., Jiang D., Jie X., Ma Y., Chen W. (2021). Facile and Green Synthesis of Bifunctional Carbon Dots for Detection of Cu^2+^ and ClO^−^ in Aqueous Solution. ACS Sustain. Chem. Eng..

[76] Xiong Y., Chen M., Mao Z., Deng Y., He J., Mu H., Li P., Zou W., Zhao Q. (2023). Synthesis of Up-Conversion Fluorescence N-Doped Carbon Dots with High Selectivity and Sensitivity for Detection of Cu^2+^ Ions. Crystals.

[77] Won S., Kim J. (2022). The Detection of Fe (III) and Ascorbic Acid by Fluorescence Quenching and Recovery of Carbon Dots Prepared from Coffee Waste. Korean J. Chem. Eng..

