# Supplementary material for: BPEI-Based N-Doped Carbon Dots with Sensitive and Selective Cu2+ Ion-Sensing Ability
Source: Micromachines (Basel). 2025 Nov 13;16(11):1275. doi: 10.3390/mi16111275 (PMC12654544; doi:10.3390/mi16111275)
Supplement: Supplementary file 1 [file micromachines-16-01275-s001.zip › micromachines-3965663-supplementary.pdf]

BPEI based N-doped carbon dots with sensitive and selective Cu^{2+} ion sensing ability

Sahin Demirci[1], Jorge H Torres[2], Nurettin Sahiner [2,3]*

[1] Department of Food Engineering, Faculty of Engineering, Istanbul Aydin University, Florya
Halit Aydin Campus, Istanbul, 34153, Turkiye.
[2] Department of Bioengineering, U.A. Whittaker College of Engineering, Florida Gulf Coast University, Fort Myers, FL 33965, USA
[3] Canakkale Onsekiz Mart University, Faculty of Engineering, Department of Chemical Engineering, Terzioglu Campus, Canakkale, 17100, Turkey.
* Correspondence: sahiner71@gmail.com; nsahiner@fgcu.edu

Table S1. Comparisons of some of the reported studies on the potential sensor applications of CDs for different metal ions.

CDs	Precursors	Synthesis method	Metal ions	LOD	Ref
N-doped	Chebulic myrobalan	Hydrothermal	Fe^{3+}	0.86 µM	[1]
P-doped	Trisodium citrate, Phosphoric acid	Hydrothermal	Fe^{3+}	9.5 nM	[2]
S-doped	Tetra (4-carboxyphenyl) porphyrin, Urea	Hydrothermal	Hg^{2+}	1.73 nM	[3]
S-doped	Succinic acid, sodium thiosulfate	Microwave irradiation	Cr^{3+}	0.17 µM	[4]
N-, and S- co-doped	Chitosan, thiourea, citric acid	Hydrothermal	Hg^{2+}	4 nM	[5]
N-, S-, and P- co-doped	Glutathione, phosphoric acid	Hydrothermal	Al^{3+}<br>Fe^{3+}	10.8 nM<br>50.7 nM	[6]
N-doped	Polyethyleneimine	Hydrothermal	Cu^{2+}	4.75 µM	[7]
N-doped	Polyethyleneimine, citric acid	Hydrothermal	Cu^{2+}	0.007 µM	[8]
N-doped	Polyethyleneimine, citric acid	Microwave irradiation	Cu^{2+}	10 µM	[9]
N-doped	Polyethyleneimine, 2,4-dihydroxybenzoic acid	Hydrothermal	Cu^{2+}	193 nM	[10]
N-doped	Polyethyleneimine, citric acid	Hydrothermal	Cu^{2+}<br>Co^{2+}	0.39 ppm<br>0.42 ppm	This study

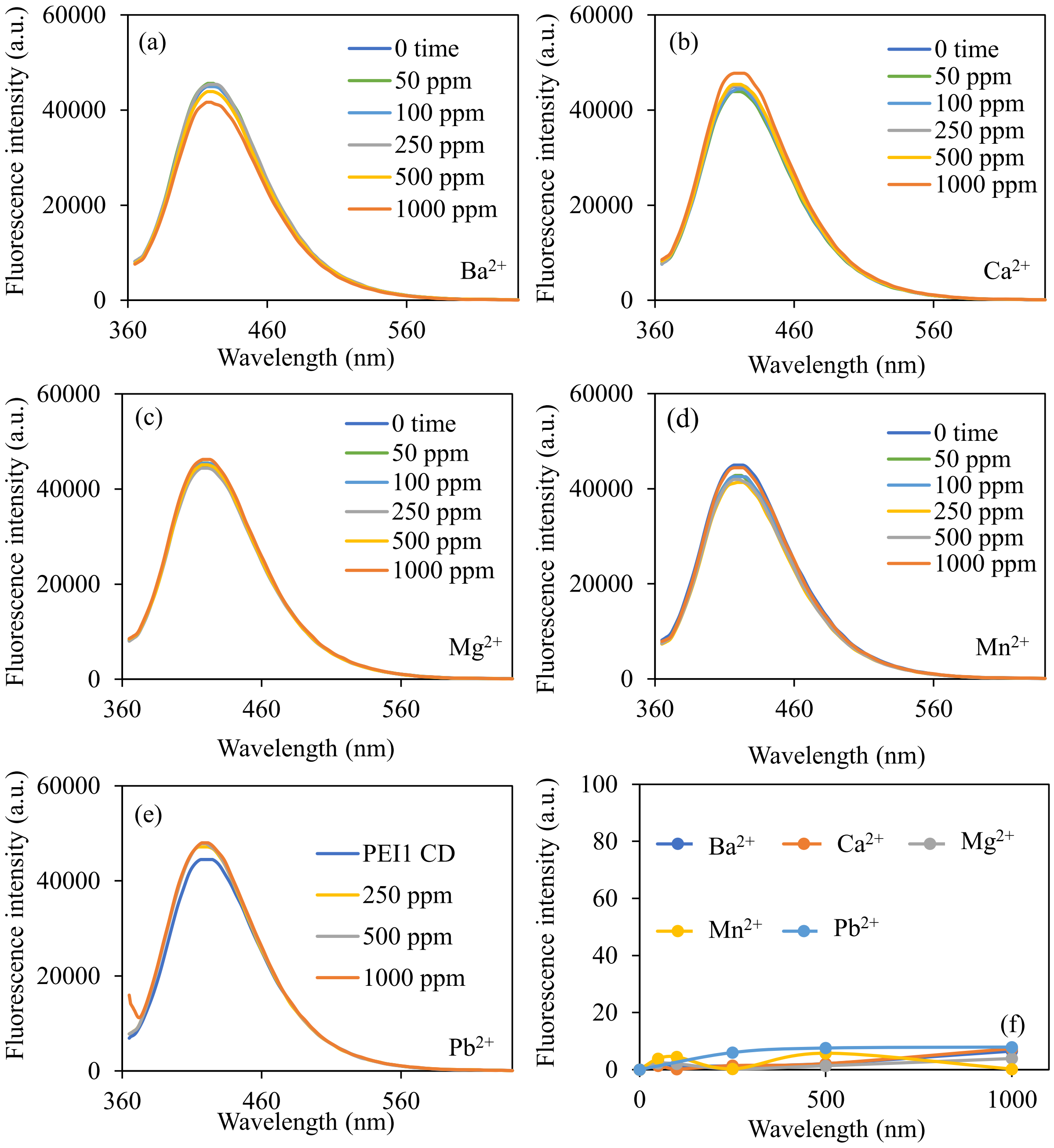

Figure S1. The fluorescence spectra of the B-PEI[1] CDs in the presence of different concentrations of (a) Ba^{2+}, (b) Ca^{2+}, (c) Mg^{2+}, (d) Mn^{2+}, (e) Pb^{2+} ions, and (f) change% in fluorescence intensity of BPEI[1] CDs in the presence of related metal ions. [Excitation wavelength for BPEI CDs: 360 nm; emission wavelengths: 420 nm; the volume of metal ion solutions is 2 mL, and final concentration of BPEI CDs is 0.6 mg/mL.]

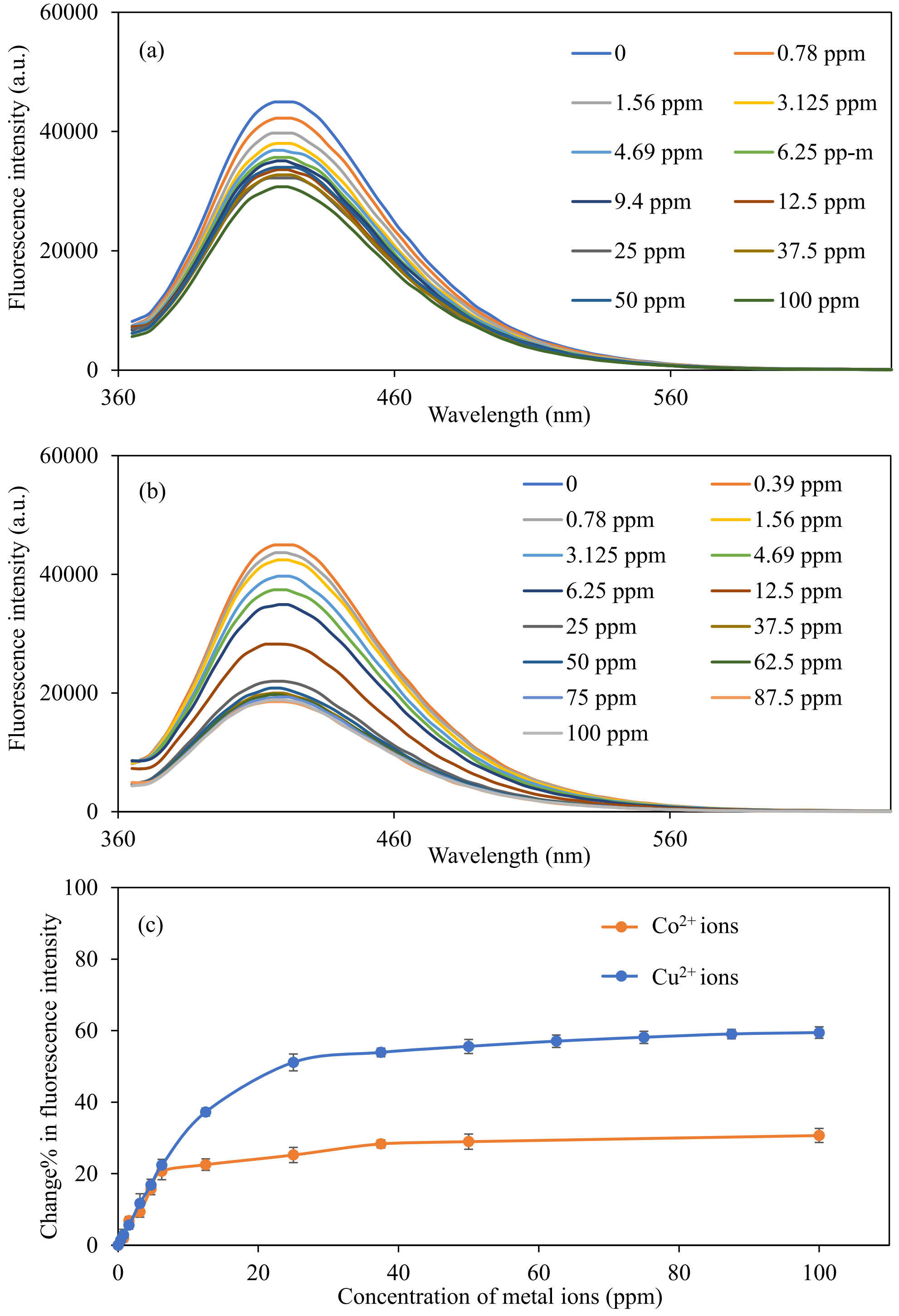

Figure S2. The fluorescence spectra of the B-PEI[1] CDs in the presence of 0-100 concentrations of (a) Co^{2+}, (b) Cu^{2+} ions, and (c) change% in fluorescence intensity of BPEI[1] CDs in the presence of related metal ions. [Excitation wavelength for BPEI CDs: 360 nm; emission wavelengths: 420 nm; the volume of metal ion solutions is 2 mL, and final concentration of BPEI CDs is 0.6 mg/mL.]

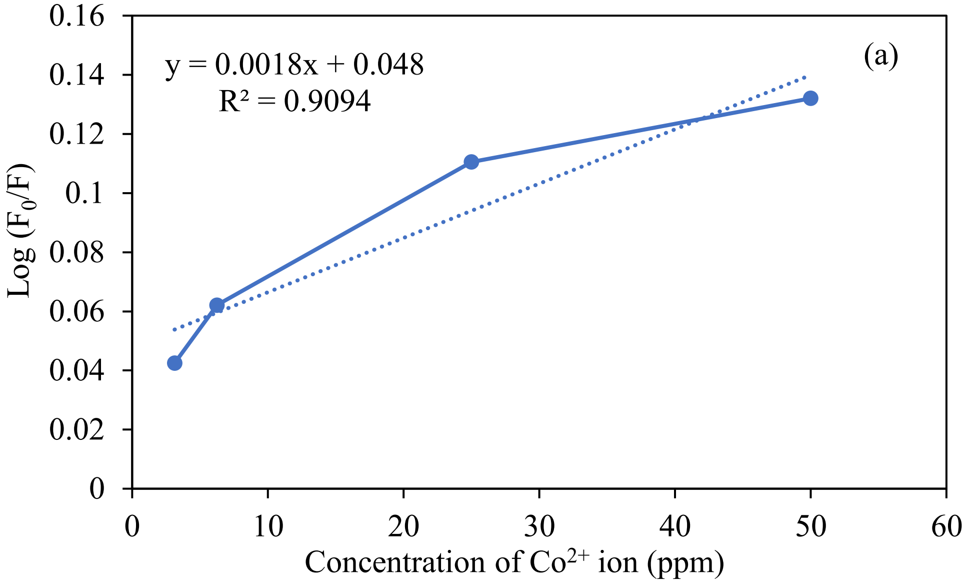

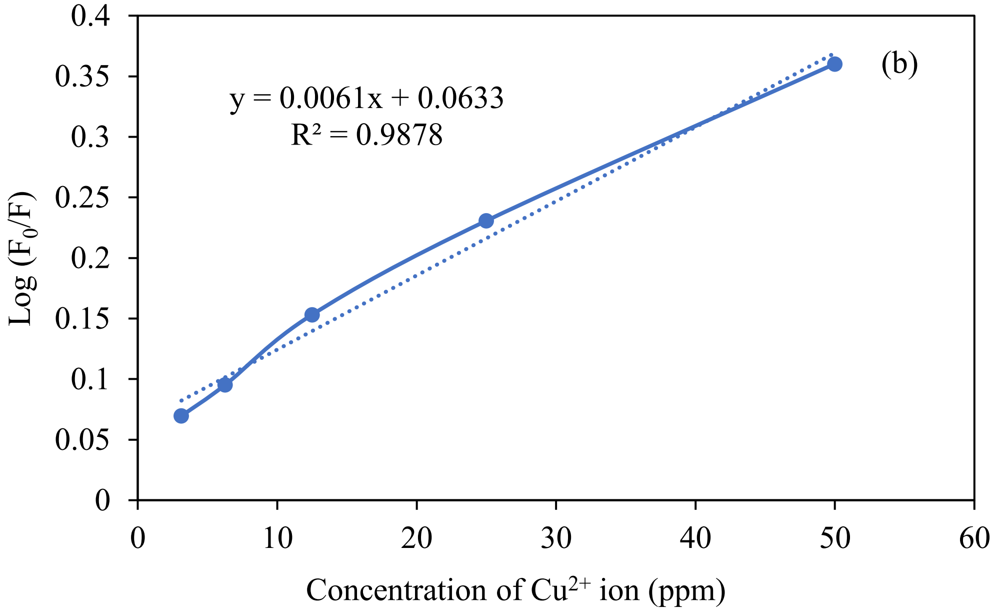

Figure S3. The linear relationship between the log(F_0/F) and the (**a**) Co^{2+}, (**b**) Cu^{2+}concentration, where F and F_0 stand for the concentrations of Cu^{2+} ions in the presence of BPEI[1] CDs.

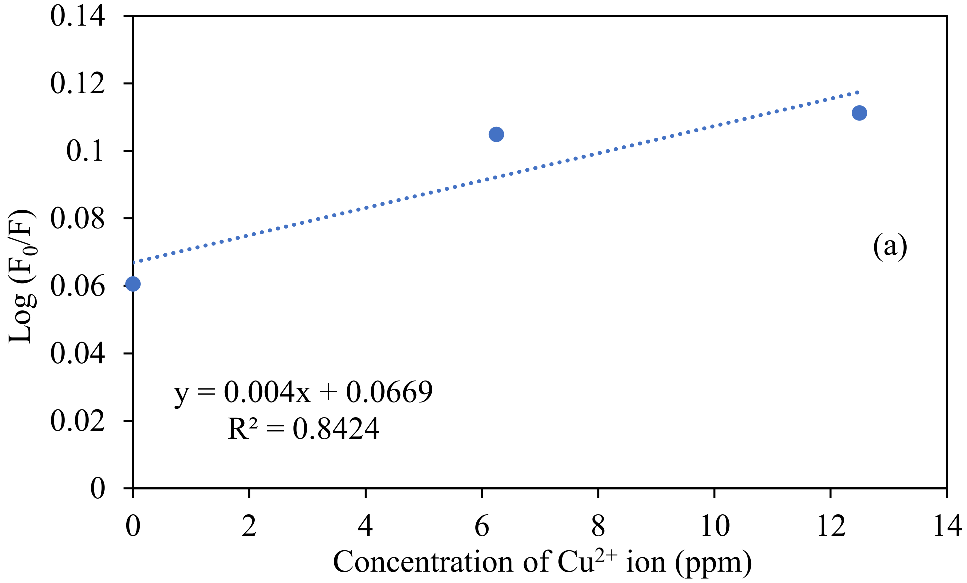

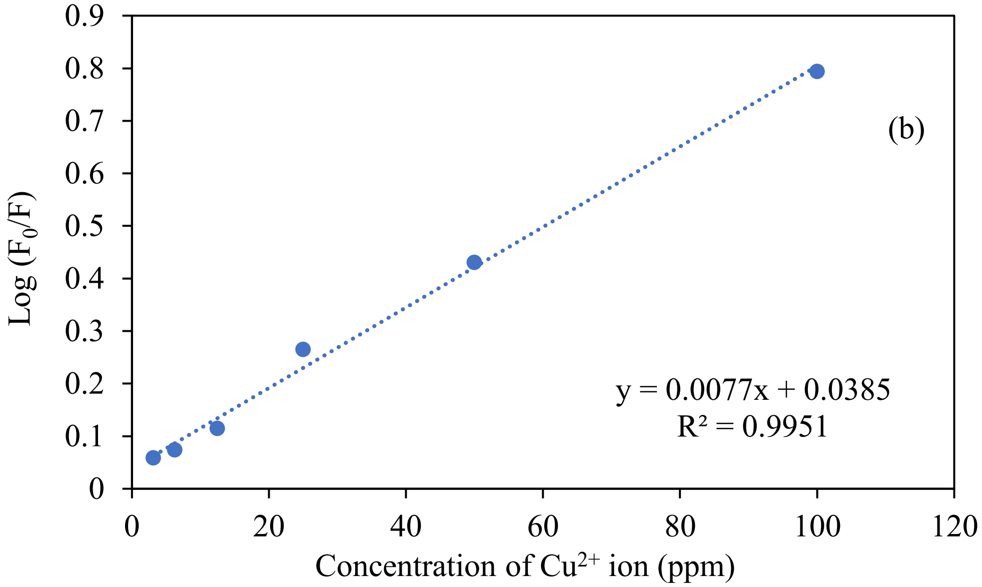

Figure S4. Figure S4: The linear relationship between the log(F_0/F) and the Cu^{2+} concentration, where F and F_0 stand for the concentrations of Cu^{2+} ions in the presence of (**a**) $BPEI^{0.5}$, and (**b**) $BPEI^2$ CDs.

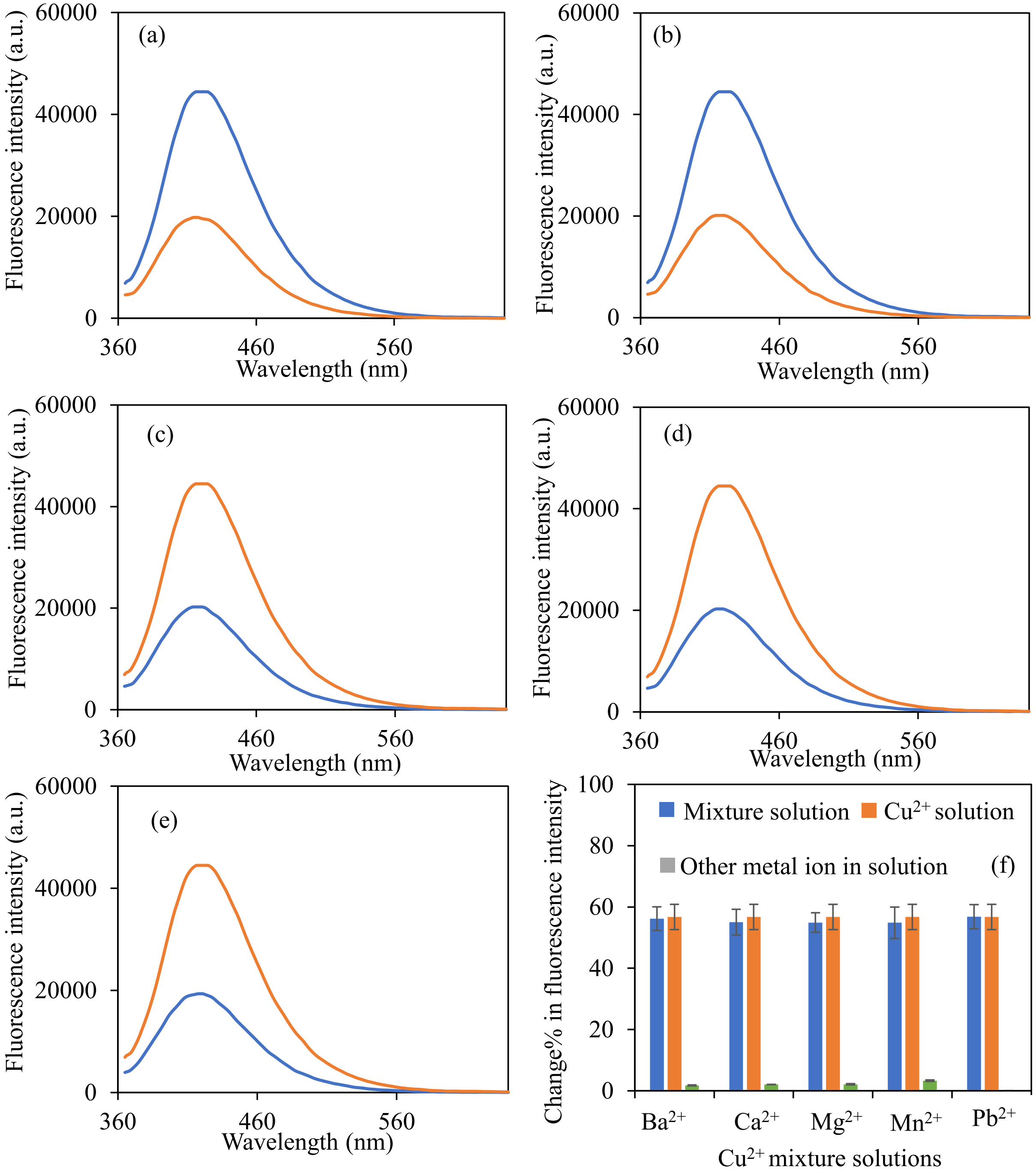

Figure S5. The fluorescence spectra of the B-PEI[1] CDs in the presence of 2 mL 50 ppm concentrations of (**a**) Cu^{2+}-Ba^{2+}, (**b**) Cu^{2+}-Ca^{2+}, (**c**) Cu^{2+}-Mg^{2+}, (**d**) Cu^{2+}-Mn^{2+}, (**e**) Cu^{2+}-Pb^{2+} mixture solution, and (f) change% in fluorescence intensity of BPEI[1] CDs in the presence of 2 mL 50 ppm mixture solutions. [Excitation wavelength for BPEI CDs: 360 nm; emission wavelengths: 420 nm; the volume of metal ion solutions is 2 mL, and the final concentration of BPEI CDs is 0.6

mg/mL. Orange-lined spectrum is bare BPEI[1] CDs in water, blue-lined spectrum is BPEI[1] CDs in mixture solutions.]

References

1. Atchudan, R.; Perumal, S.; Edison, T.N.J.I.; Sundramoorthy, A.K.; Vinodh, R.; Sangaraju, S.; Kishore, S.C.; Lee, Y.R. Natural Nitrogen-Doped Carbon Dots Obtained from Hydrothermal Carbonization of Chebulic Myrobalan and Their Sensing Ability toward Heavy Metal Ions. *Sensors* 2023, *23*, 787, doi:10.3390/s23020787.

2. Kalaiyarasan, G.; Joseph, J.; Kumar, P. Phosphorus-Doped Carbon Quantum Dots as Fluorometric Probes for Iron Detection. *ACS Omega* 2020, *5*, 22278–22288, doi:10.1021/acsomega.0c02627.

3. Zeng, J.; Liao, L.; Lin, X.; Liu, G.; Luo, X.; Luo, M.; Wu, F. Red-Emissive Sulfur-Doped Carbon Dots for Selective and Sensitive Detection of Mercury (II) Ion and Glutathione. *Int J Mol Sci* 2022, *23*, 9213, doi:10.3390/ijms23169213.

4. Kamali, S.R.; Chen, C.-N.; Agrawal, D.C.; Wei, T.-H. Sulfur-Doped Carbon Dots Synthesis under Microwave Irradiation as Turn-off Fluorescent Sensor for Cr(III). *J Anal Sci Technol* 2021, *12*, 48, doi:10.1186/s40543-021-00298-y.

5. Chaghaghazardi, M.; Kashanian, S.; Nazari, M.; Omidfar, K.; Joseph, Y.; Rahimi, P. Nitrogen and Sulfur Co-Doped Carbon Quantum Dots Fluorescence Quenching Assay for Detection of Mercury (II). *Spectrochim Acta A Mol Biomol Spectrosc* 2023, *293*, 122448, doi:10.1016/j.saa.2023.122448.

6. Mohandoss, S.; Ganesan, S.; Palanisamy, S.; You, S.; Velsankar, K.; Sudhahar, S.; Lo, H.-M.; Lee, Y.R. Nitrogen, Sulfur, and Phosphorus Co-Doped Carbon Dots-Based Ratiometric Chemosensor for Highly Selective Sequential Detection of Al3+ and Fe3+ Ions in Logic Gate, Cell Imaging, and Real Sample Analysis. *Chemosphere* 2023, *313*, 137444, doi:10.1016/j.chemosphere.2022.137444.

7. Liu, X.; Zhang, S.; Xu, H.; Wang, R.; Dong, L.; Gao, S.; Tang, B.; Fang, W.; Hou, F.; Zhong, L.; et al. Nitrogen-Doped Carbon Quantum Dots from Poly(Ethyleneimine) for Optical Dual-Mode Determination of Cu 2+ and <scp>l</Scp> -Cysteine and Their Logic Gate Operation. *ACS Appl Mater Interfaces* 2020, *12*, 47245–47255, doi:10.1021/acsami.0c12750.

8. Chen, Z.; Han, X.; Lin, Z.; Fan, Y.; Shi, G.; Zhang, S.; Zhang, M. Facile Reflux Synthesis of Polyethyleneimine-capped Fluorescent Carbon Dots for Sequential Bioassays toward Cu 2+ /H 2 S and Its Application for a Logic System. *Biotechnol Appl Biochem* 2019, *66*, 426–433, doi:10.1002/bab.1739.

9. Hu, G.; Pei, Z.; Shen, B.; Li, Y.; Wei, W.; Zhang, J.; Li, J. Correlation between Surface Structure of Carbon Dots and Selective Detection of Heavy Metal Ions. *Applied Physics A* 2024, *130*, 122, doi:10.1007/s00339-023-07265-x.

10. Yang, J.; Jin, X.; Cheng, Z.; Zhou, H.; Gao, L.; Jiang, D.; Jie, X.; Ma, Y.; Chen, W. Facile and Green Synthesis of Bifunctional Carbon Dots for Detection of Cu 2+ and ClO – in Aqueous Solution. *ACS Sustain Chem Eng* 2021, *9*, 13206–13214, doi:10.1021/acssuschemeng.1c03868.
